# Supplementary material for: Understanding the role of media in the formation of public sentiment towards the police
Source: Commun Psychol. 2024 Feb 7;2:11. doi: 10.1038/s44271-024-00059-8 (PMC11332102; doi:10.1038/s44271-024-00059-8)
Supplement: Supplementary file 2 — Supplementary Information [file 44271_2024_59_MOESM2_ESM.pdf]

Understanding the role of media in the formation of public sentiment towards the police
Supplementary Information

Rayan Succar[1,2], Salvador Ramallo[1,3], Rishita Das[1,2], Roni Barak Ventura[1,2], and Maurizio Porfiri[1,2,4,*]

[1]Center for Urban Science and Progress, New York University, Brooklyn, New York, 11201, USA
[2]Department of Mechanical and Aerospace Engineering, Tandon School of Engineering, New York University, Brooklyn, New York, 11201, USA
[3]Department of Quantitative Methods, University of Murcia, Murcia, 30100, Spain
[4]Department of Biomedical Engineering, Tandon School of Engineering, New York University, Brooklyn, New York, 11201, USA
[*]Corresponding author: Maurizio Porfiri, mporfiri@nyu.edu

Supplementary Note 1 Data curation of media coverage

In the present study, all media coverage data were collected from the ProQuest database by scraping for news articles that contained the chosen keywords from a selection of newspapers. In particular, the selection of newspapers to create the time series of police brutality was based on the available newspapers in the database from the top 20 newspapers by circulation in the US according to Turvill (2018). Specifically, we considered the following newspapers, sorted from the most to the least circulated: Wall Street Journal, New York Times, USA Today, Washington Post, Los Angeles Times, Star Tribune, Tampa Bay Times, Chicago Tribune, Newsday, Boston Globe, Houston Chronicle, Arizona Republic, Philadelphia Inquirer, Dallas Morning News, San Francisco Chronicle, Buffalo News, and New York Daily News. With respect to media coverage from Twitter accounts of newspapers, the top 10 most-followed newspapers accounts (Cision, 2018) were used. Specifically, we considered the following newspapers, ordered from the one with the most to the one with the least number of followers: The New York Times (@nytimes), The Wall Street Journal (@WSJ), The Washington Post (@washingtonpost), USA Today (@USATODAY), Los Angeles Times (@latimes), New York Post (@nypost), Atlanta Journal-Constitution (@ajc), Chicago Tribune (@chicagotribune), The Boston Globe (@BostonGlobe), New York Daily News (@NYDailyNews).

In order to create the time series of media coverage of local crime, we utilized newspapers in ProQuest from the top 20 by circulation in the states in which the metropolitan area was located, according to OfficialUSA (2022). Note that in some states, the ProQuest databases did not contain all top 20 newspapers and that some metropolitan areas cross the borders of multiple states. The queries inputted to search the database were composed of the word "crime" either in the text or in the headline, together with the names of any of the cities in the metropolitan area with at least 100,000 residents (Supplementary Table 1).

Supplementary Note 2 Data

Here, we report daily data for media coverage of local crime, number of positive tweets about the police at a daily resolution, number of negative tweets about the police at a daily resolution, and number of negative tweets at the resolution of one minute in the wake of George Floyd's murder for all the 18 metropolitan areas, excluding New York City that is presented in the main document.

Metropolitan area	Cities	Newspapers
Atlanta	Atlanta, Sandy Springs, South Fulton	The Atlanta Journal-Constitution,<br>Savannah Morning News
Baltimore	Baltimore, Columbia	The Baltimore Sun, Cumberland Times-News,<br>Carroll County Times
Boston	Boston, Worcester, Providence, Lowell,<br>Cambridge, Quincy, Manchester	Boston Globe, Boston Herald,<br>The Berkshire Eagle
Chicago	Chicago, Aurora, Joliet, Naperville, Elgin	Chicago Tribune, Chicago Sun-Times,<br>State Journal Register, The Belleville News - Democrat,<br>Quincy Herald - Whig
Dallas	Dallas, Fort Worth, Arlington,<br>McKinney, Grand Prairie,<br>Frisco, Carrollton,<br>Denton, Mesquite, Richardson,<br>Lewisville, Allen	The Dallas Morning News, Houston Chronicle,<br>Fort Worth Star - Telegram, San Antonio Express-News,<br>Austin American Statesman, Corpus Christi Caller - Times,<br>Waco Tribune - Herald, El Paso Times, Victoria Advocate,<br>Abilene Reporter-News, Longview News - Journal,<br>Lubbock Avalanche - Journal
Denver	Denver, Arvada, Aurora, Centennial,<br>Highlands Ranch,<br>Lakewood, Thornton, Westmister	Denver Post, Colorado Springs Gazette,<br>Pueblo Chieftain, Fort Collins Coloradoan
Detroit	Detroit, Warren, Sterling Heights,	Detroit Free Press, Lansing State Journal,
Houston	Dearborn, Clinton<br>Houston, The Woodlands, Sugar Land	The Dallas Morning News, Houston Chronicle,<br>San Antonio Express-News, Austin American Statesman,<br>Corpus Christi Caller - Times, Waco Tribune - Herald,<br>El Paso Times, Victoria Advocate, Abilene Reporter-News,<br>Longview News - Journal, Lubbock Avalanche - Journal<br>Fort Worth Star - Telegram, Battle Creek Enquirer
Los Angeles	Los Angeles, Long Beach, Santa Clarita,<br>Lancaster, Palmdale, Pomona,<br>Downey, West Covina, El Monte,<br>Norwalk, Anaheim, Santa Ana,<br>Irvine, Garden Grove, Inglewood, Torrance,<br>Fullerton, Orange, Costa Mesa, Burbank<br>Glendale, Pasadena,	Los Angeles Times, East Bay Times,<br>San Francisco Chronicle, Orange County Register,<br>San Jose Mercury News, The Fresno Bee,<br>Ventura County Star, Modesto Bee,<br>Marin Independent Journal<br>San Gabriel Valley Tribune, The Sacramento Bee,<br>The San Diego Union - Tribune, Torrance Daily Breeze
Miami	Miami, Hialeah, Fort Lauderdale,<br>Hollywood, Miramar, Coral Springs,<br>Pompano Beach, West Palm Beach, Davie<br>Miami Gardens, Pembroke Pines,	Tampa Bay Times, Orlando Sentinel, Palm Beach Post,<br>Sarasota Herald Tribune, Florida Times Union,<br>The Daytona Beach News - Journal, Gainesville Sun<br>Miami Herald
Minneapolis	Minneapolis, Saint Paul	Star Tribune
New York City	New York City, Yonkers, Woodbridge, New Haven,<br>Stamford, Smithtown, Paterson,<br>North Hempstead, Newark,<br>Huntington, Hempstead, Elizabeth,<br>Waterbury, Oyster Bay,<br>Edison, Brookhaven, Jersey City, Islip,<br>Bridgeport, Babylon	Philadelphia Inquirer, Pittsburgh Post - Gazette,<br>New York Times, New York Daily News, Newsday,<br>Rochester Democrat and Chronicle, Asbury Park Press,<br>Press of Atlantic City, Hartford Courant<br>Buffalo News, Pittsburgh Tribune - Review,<br>Home News Tribune
Philadelphia	Philadelphia	LNP, York Daily Record
Phoenix	Phoenix, Mesam Chandler, Glendale,<br>Scottsdale, Gilbert, Tempe, Preoria	Arizona Republic, Arizona Daily Star
Riverside	San Bernardino, Riverside,<br>Rancho Cucamonga, Ontario,<br>Murrieta, Temecula<br>Moreno Valley, Fontana,<br>Corona, Victorville	Los Angeles Times, East Bay Times,<br>San Francisco Chronicle, Orange County Register,<br>San Jose Mercury News, The Fresno Bee,<br>Ventura County Star, Modesto Bee,<br>Marin Independent Journal<br>San Gabriel Valley Tribune, The Sacramento Bee,<br>The San Diego Union - Tribune, Torrance Daily Breeze
San Francisco	San Francisco, Oakland, San Jose,<br>Hayward, Sunnyvale, Concord,<br>Berkeley, Fairfield, Antioch,<br>San Mateo, Vacaville<br>Fremont, Santa Rosa,<br>Santa Clara, Vallejo,<br>Richmond, Daly City	Los Angeles Times, East Bay Times,<br>San Francisco Chronicle, Orange County Register,<br>San Jose Mercury News, The Fresno Bee,<br>Ventura County Star, Modesto Bee,<br>Marin Independent Journal<br>San Gabriel Valley Tribune, The Sacramento Bee,<br>The San Diego Union - Tribune, Torrance Daily Breeze
Tampa	Tampa Bay, St. Petersburg,<br>Riverview, Brandon, Spring Hill<br>Clearwater, Lakeland,	Tampa Bay Times, Orlando Sentinel, Palm Beach Post,<br>Sarasota Herald Tribune, Florida Times Union,<br>The Daytona Beach News - Journal, Gainesville Sun<br>Miami Herald
Washington, D.C.	Washington, Arlington, Alexandria	The Washington Post, Washington Examiner

Supplementary Table 1: Details of the ProQuest search on media coverage of local crime. Cities with more than 100,000 residents in the chosen 18 metropolitan areas, along with the most circulated newspapers from the states where the metropolitan area is.

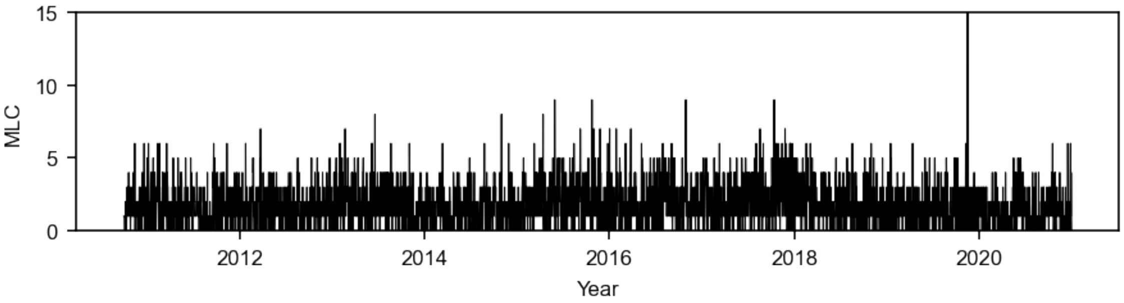

(a)

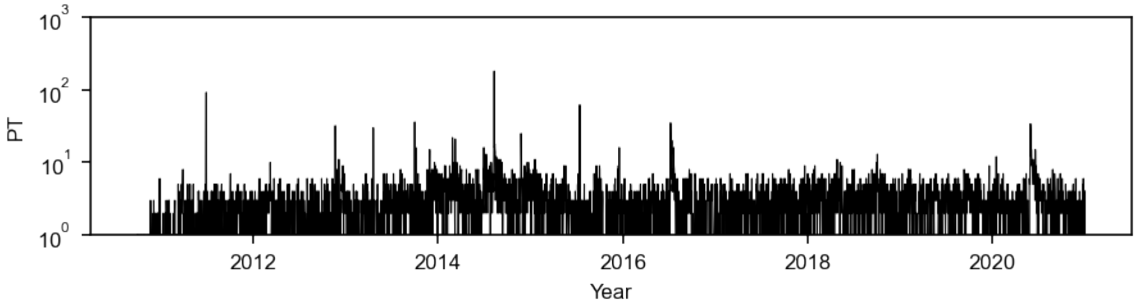

(b)

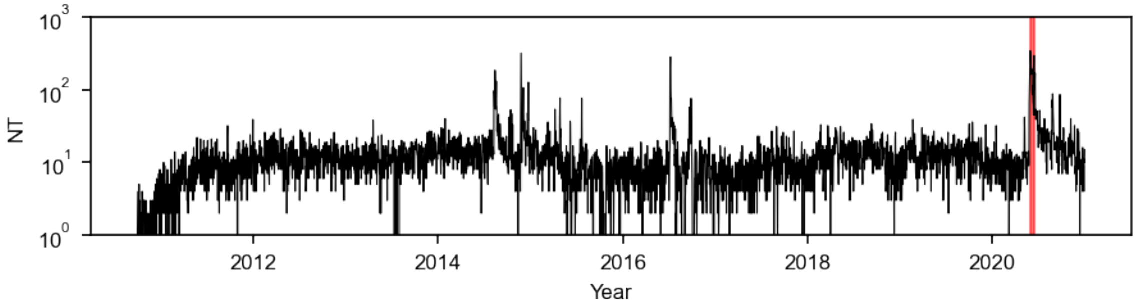

(c)

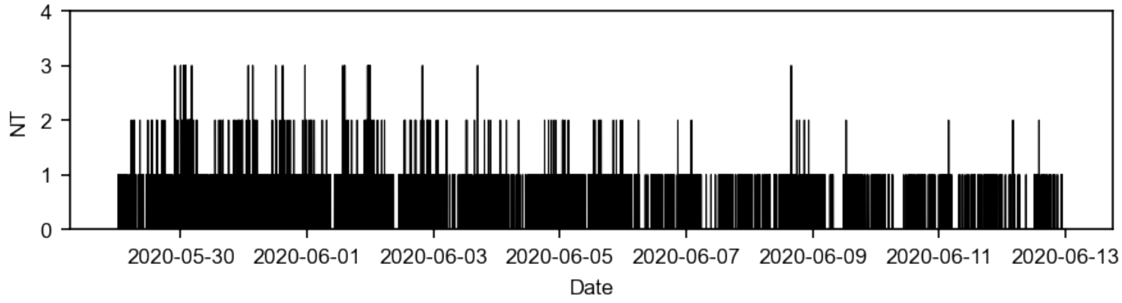

(d)

Supplementary Figure 1: Time series for the Atlanta metropolitan area. (a) Daily media coverage of local crime (MLC), with a peak of 15 articles registered on November 16, 2019. (b) Daily number of positive tweets (PT) about the police, with a peak of 181 registered on August 11, 2014. (c) Daily number of negative tweets (NT) about the police, with a peak of 344 registered on May 30, 2020; activity in the wake of George Floyd's murder is highlighted in red. (d) Zoomed-in view at the resolution of one minute of the number of negative tweets about to the police in the wake of George Floyd's murder period, from May 29, 2020 until June 13, 2020.

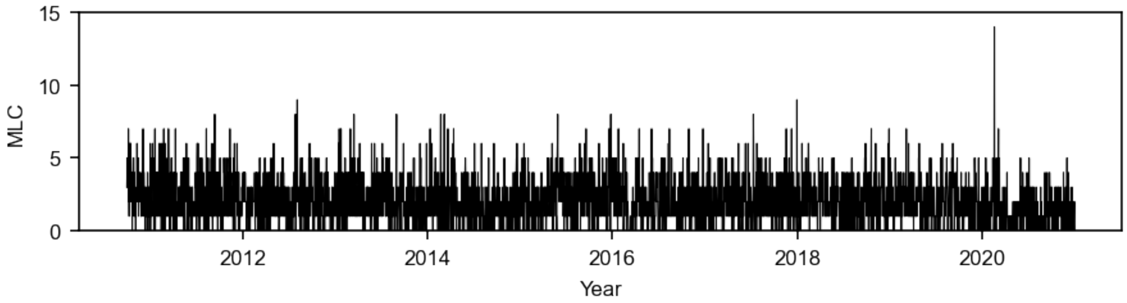

(a)

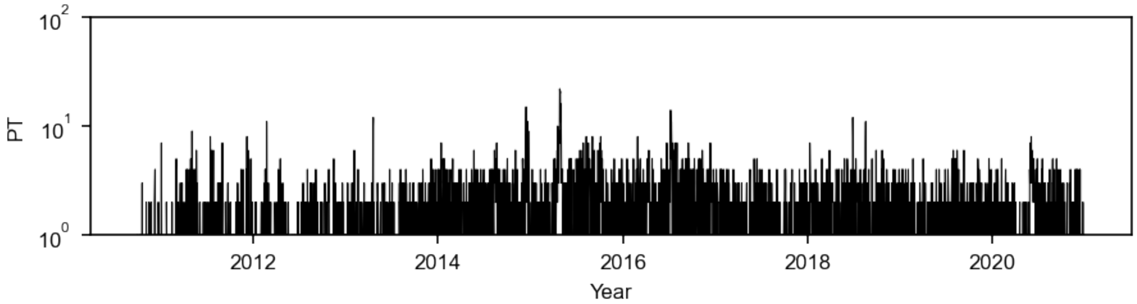

(b)

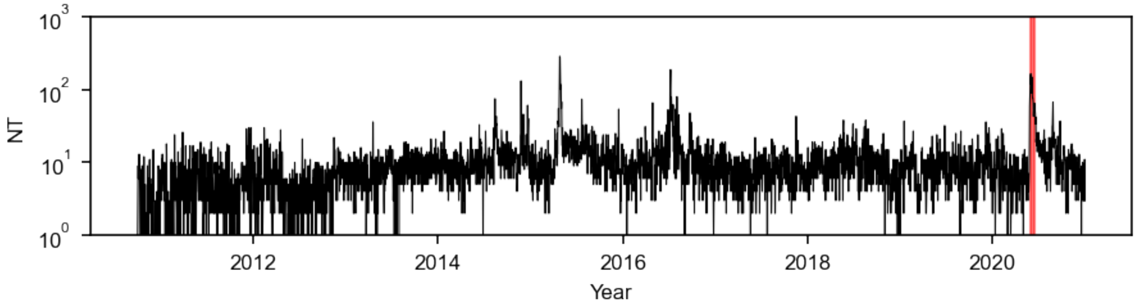

(c)

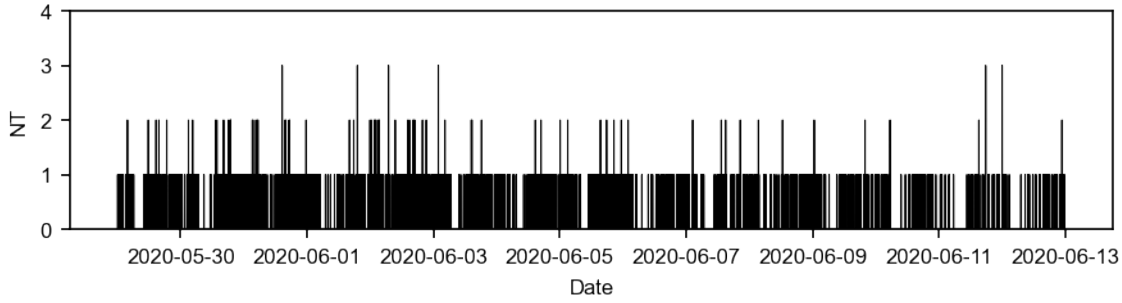

(d)

Supplementary Figure 2: Time series for the Baltimore metropolitan area. (a) Daily media coverage of local crime (MLC), with a peak of 14 articles registered on February 18, 2020. (b) Daily number of positive tweets (PT) about the police, with a peak of 22 registered on April 27, 2015. (c) Daily number of negative tweets (NT) about the police, with a peak of 287 registered on April 27, 2015; activity in the wake of George Floyd's murder is highlighted in red. (d) Zoomed-in view at the resolution of one minute of the number of negative tweets about to the police in the wake of George Floyd's murder period, from May 29, 2020 until June 13, 2020.

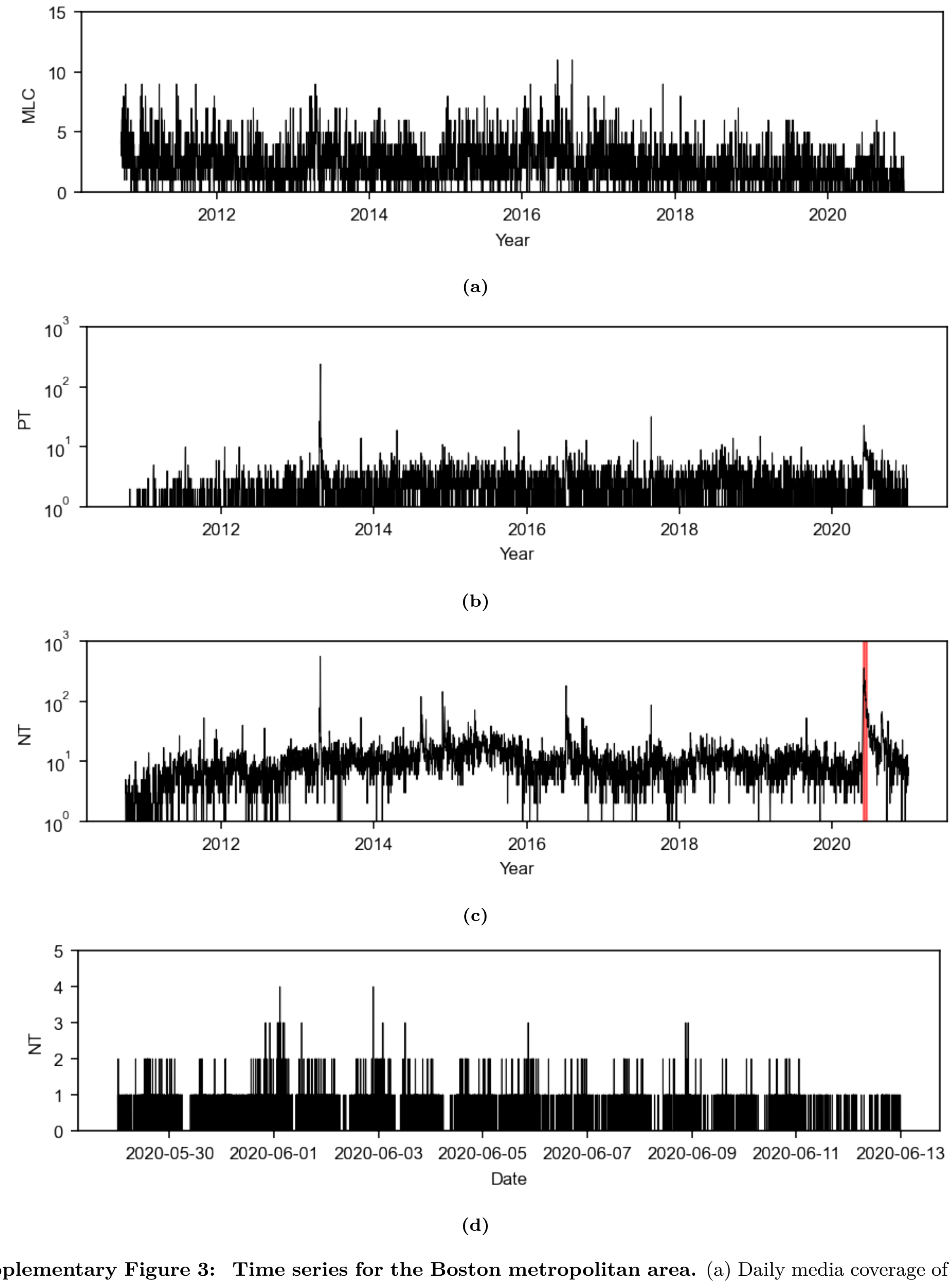

Supplementary Figure 3: Time series for the Boston metropolitan area. (a) Daily media coverage of local crime (MLC), with a peak of 11 articles registered on June 18, 2016. (b) Daily number of positive tweets (PT) about the police, with a peak of 241 registered on April 20, 2013. (c) Daily number of negative tweets (NT) about the police, with a peak of 558 registered on April 19, 2013; activity in the wake of George Floyd's murder is highlighted in red. (d) Zoomed-in view at the resolution of one minute of the number of negative tweets about to the police in the wake of George Floyd's murder period, from May 29, 2020 until June 13, 2020.

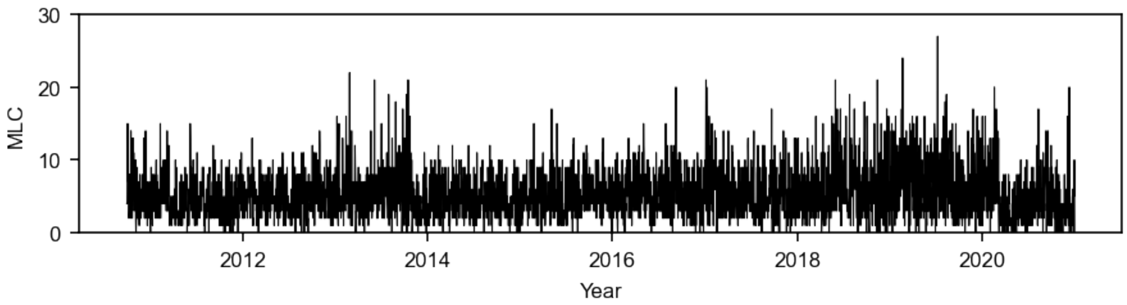

(a)

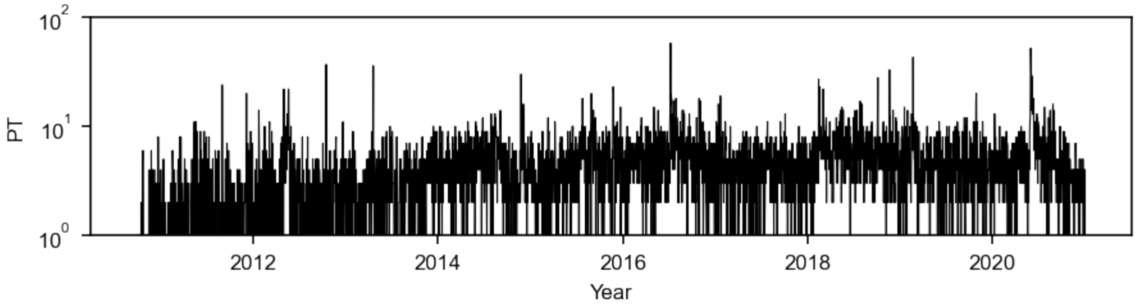

(b)

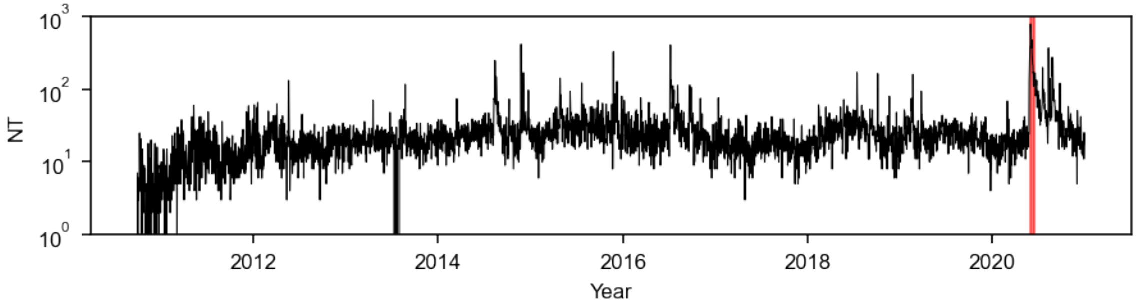

(c)

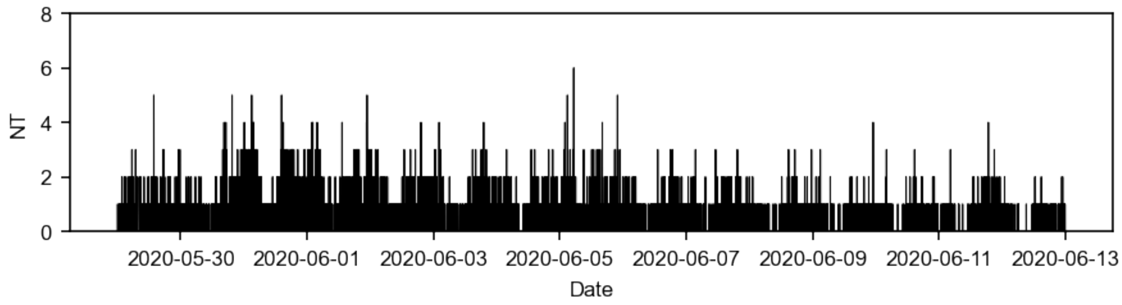

(d)

Supplementary Figure 4: Time series for the Chicago metropolitan area. (a) Daily media coverage of local crime (MLC), with a peak of 27 articles registered on July 9, 2019. (b) Daily number of positive tweets (PT) about the police, with a peak of 58 registered on July 8, 2016. (c) Daily number of negative tweets (NT) about the police, with a peak of 792 registered on May 31, 2020; activity in the wake of George Floyd's murder is highlighted in red. (d) Zoomed-in view at the resolution of one minute of the number of negative tweets about to the police in the wake of George Floyd's murder period, from May 29, 2020 until June 13, 2020.

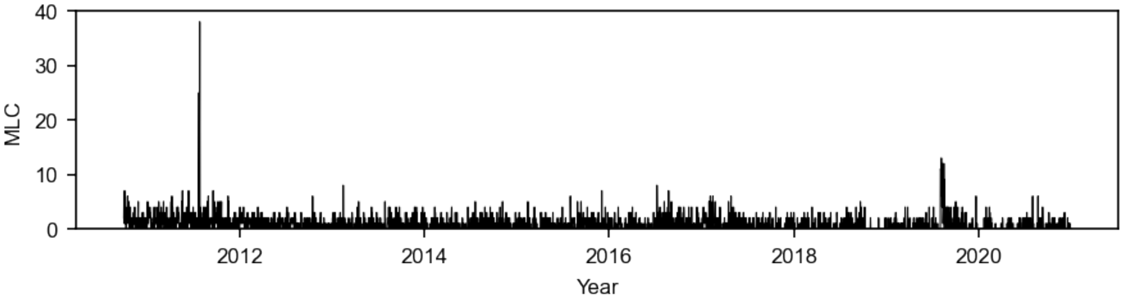

(a)

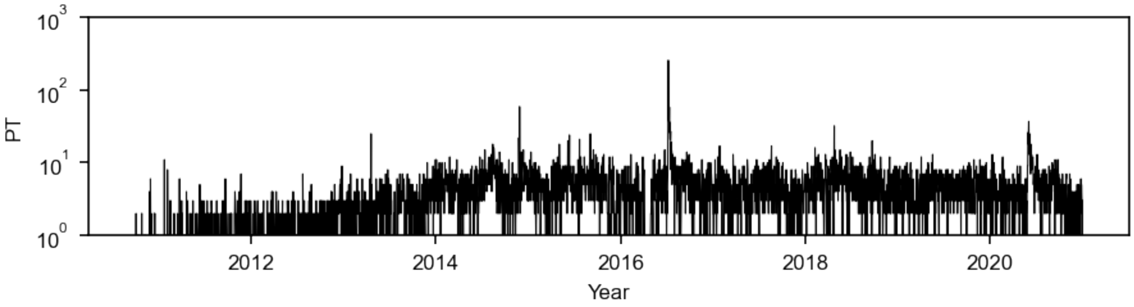

(b)

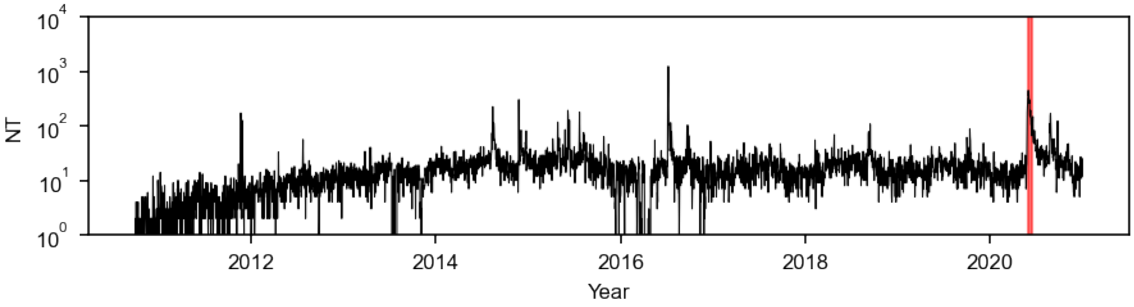

(c)

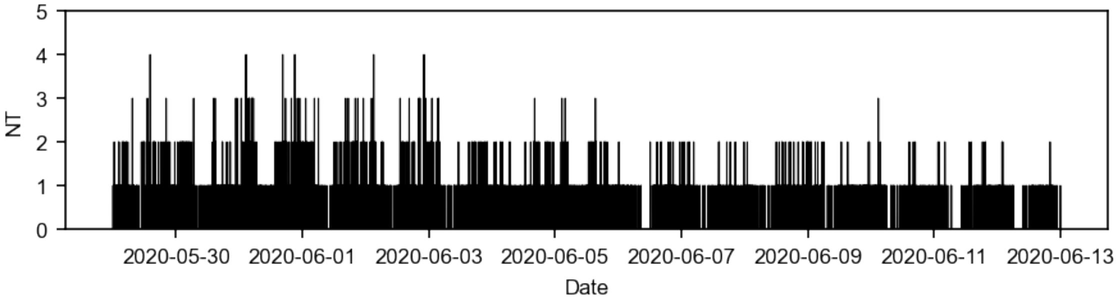

(d)

Supplementary Figure 5: Time series for the Dallas metropolitan area. (a) Daily media coverage of local crime (MLC), with a peak of 38 articles registered on July 28, 2011. (b) Daily number of positive tweets (PT) about the police, with a peak of 256 registered on July 8, 2016. (c) Daily number of negative tweets (NT) about the police, with a peak of 1,235 registered on July 8, 2016; activity in the wake of George Floyd's murder is highlighted in red. (d) Zoomed-in view at the resolution of one minute of the number of negative tweets about to the police in the wake of George Floyd's murder period, from May 29, 2020 until June 13, 2020.

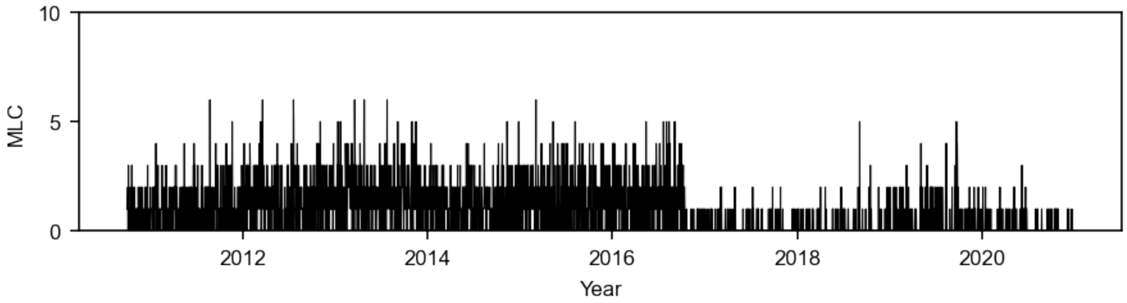

(a)

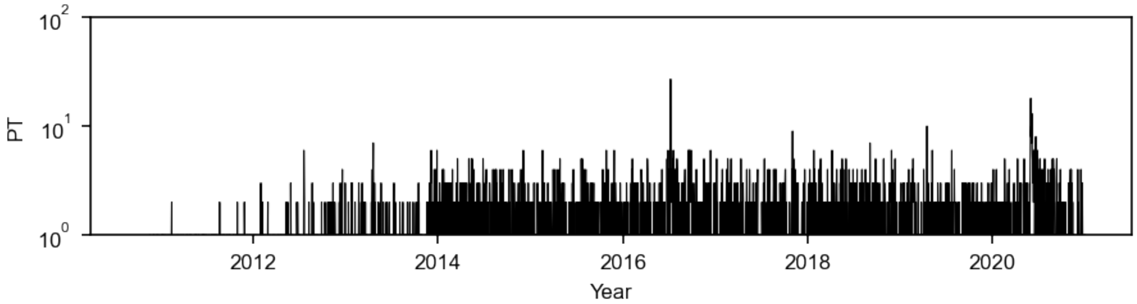

(b)

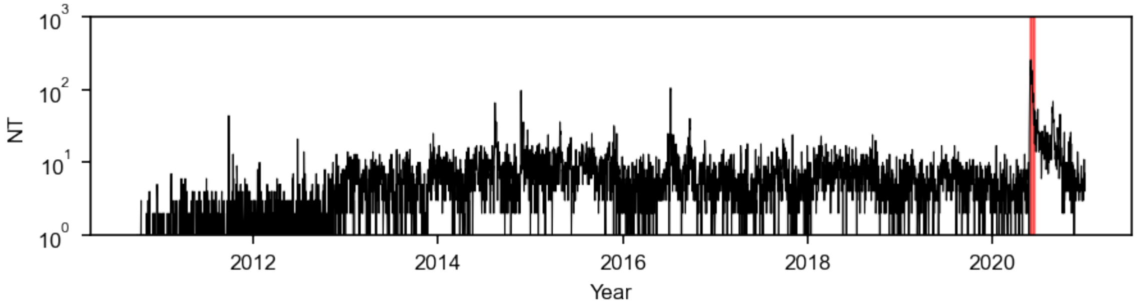

(c)

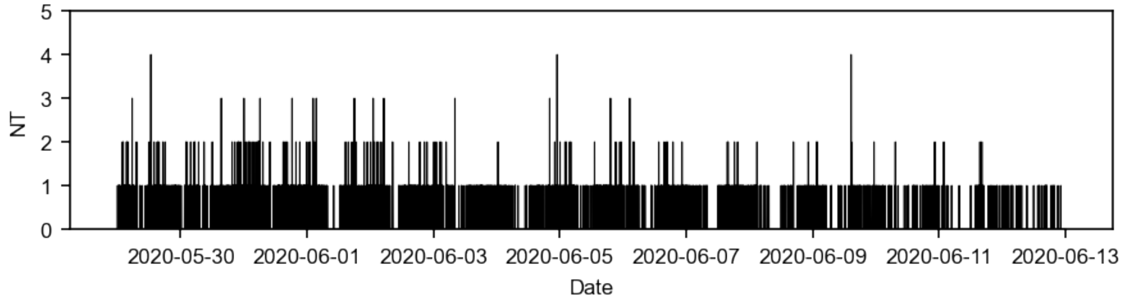

(d)

Supplementary Figure 6: Time series for the Denver metropolitan area. (a) Daily media coverage of local crime (MLC), with a peak of 6 articles registered on August 25, 2011. (b) Daily number of positive tweets (PT) about the police, with a peak of 27 registered on May 31, 2020July 8, 2016. (c) Daily number of negative tweets (NT) about the police, with a peak of 256 registered on May 31, 2020; activity in the wake of George Floyd's murder is highlighted in red. (d) Zoomed-in view at the resolution of one minute of the number of negative tweets about to the police in the wake of George Floyd's murder period, from May 29, 2020 until June 13, 2020.

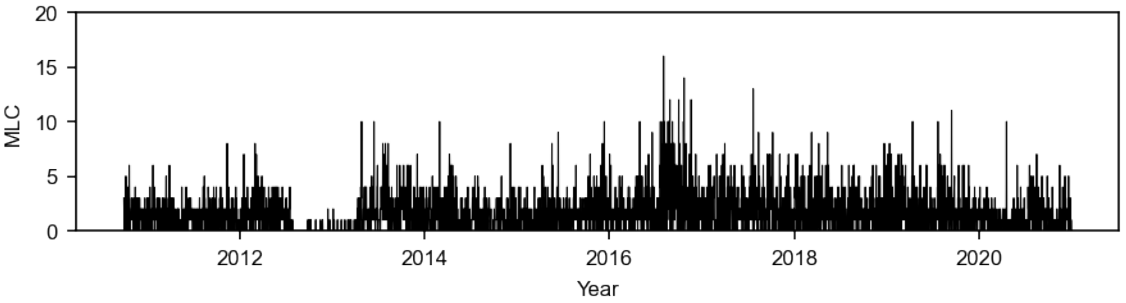

(a)

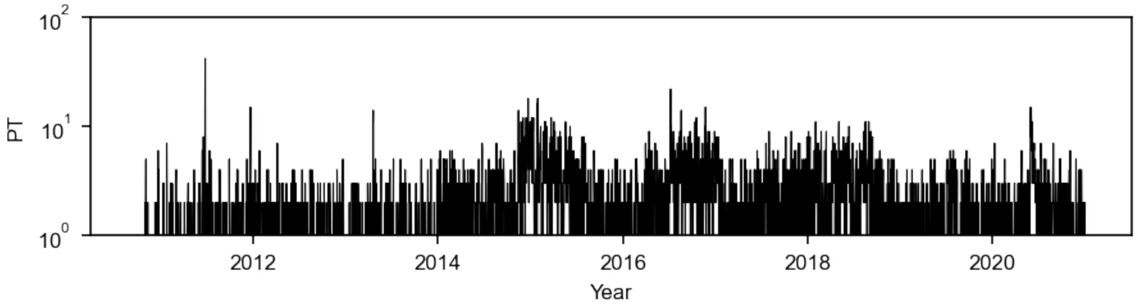

(b)

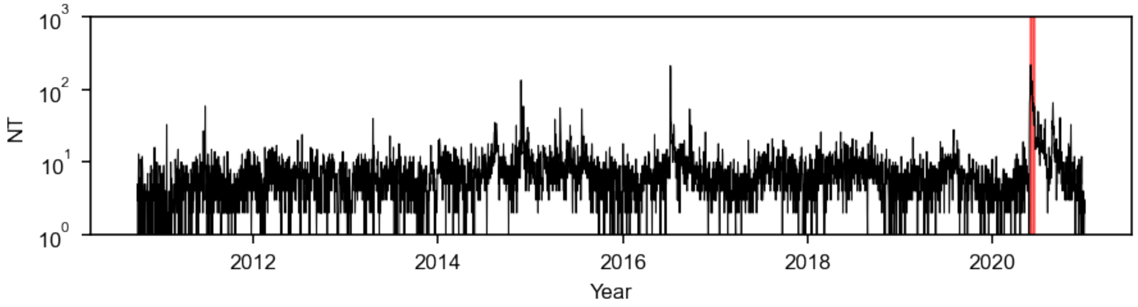

(c)

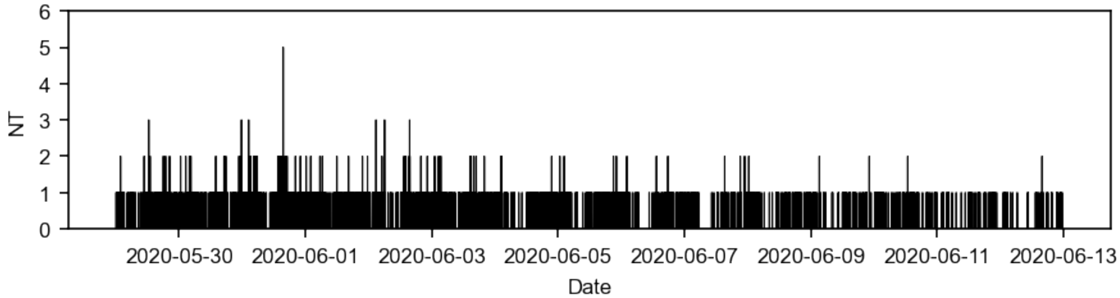

(d)

Supplementary Figure 7: Time series for the Detroit metropolitan area. (a) Daily media coverage of local crime (MLC), with a peak of 16 articles registered on August 2, 2016. (b) Daily number of positive tweets (PT) about the police, with a peak of 42 registered on June 26, 2011. (c) Daily number of negative tweets (NT) about the police, with a peak of 218 registered on May 31, 2020; activity in the wake of George Floyd's murder is highlighted in red. (d) Zoomed-in view at the resolution of one minute of the number of negative tweets about to the police in the wake of George Floyd's murder period, from May 29, 2020 until June 13, 2020.

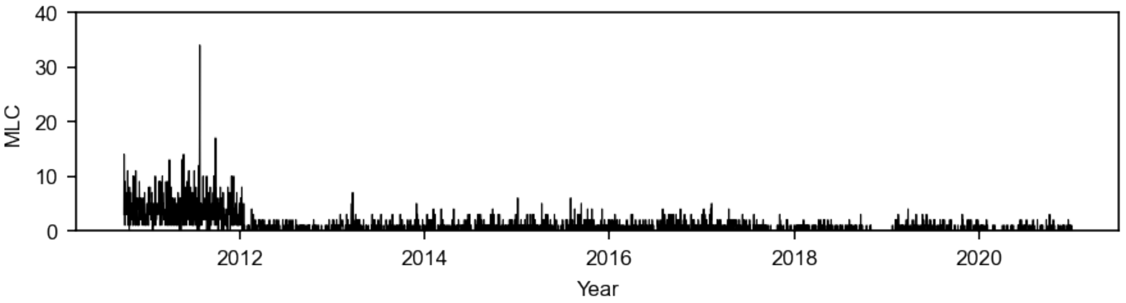

(a)

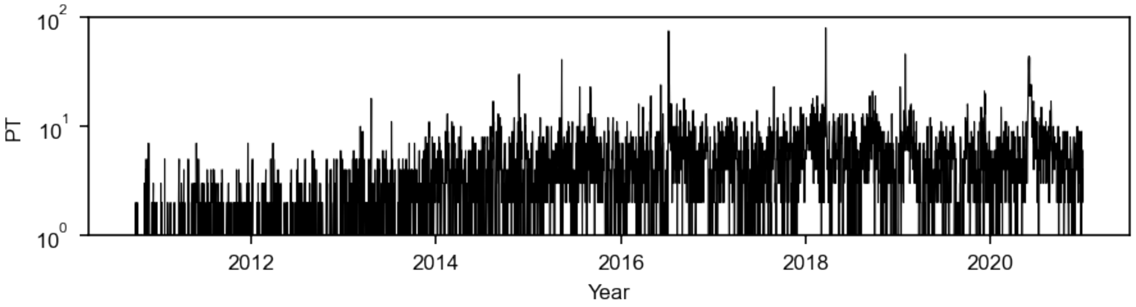

(b)

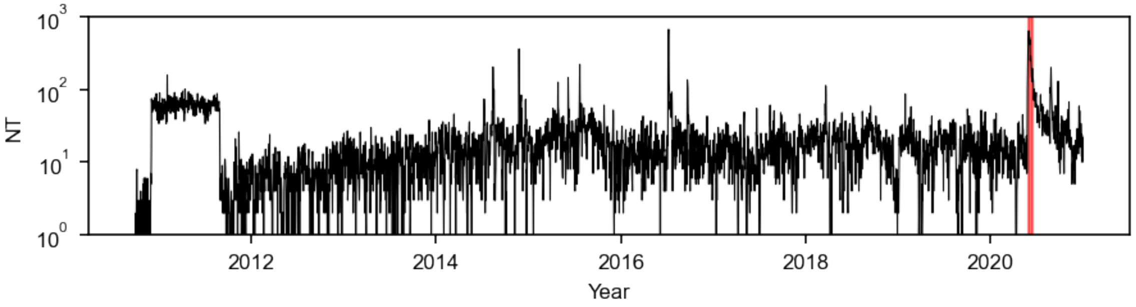

(c)

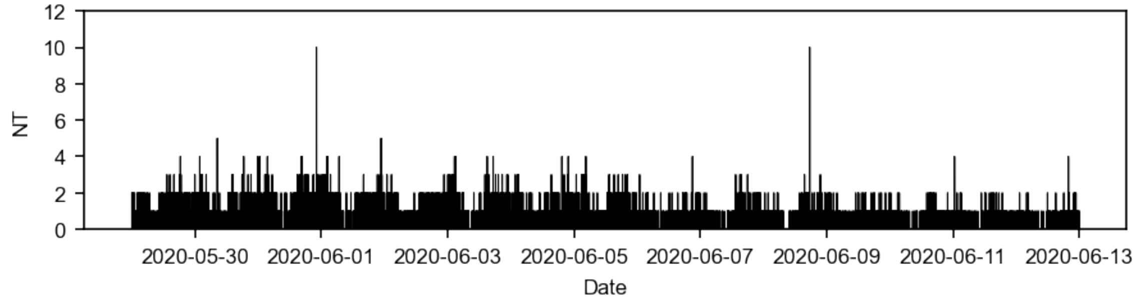

(d)

Supplementary Figure 8: Time series for the Houston metropolitan area. (a) Daily media coverage of local crime (MLC), with a peak of 34 articles registered on July 28, 2011. (b) Daily number of positive tweets (PT) about the police, with a peak of 80 registered on March 21, 2018. (c) Daily number of negative tweets (NT) about the police, with a peak of 674 registered on July 8, 2016; activity in the wake of George Floyd's murder is highlighted in red. (d) Zoomed-in view at the resolution of one minute of the number of negative tweets about to the police in the wake of George Floyd's murder period, from May 29, 2020 until June 13, 2020.

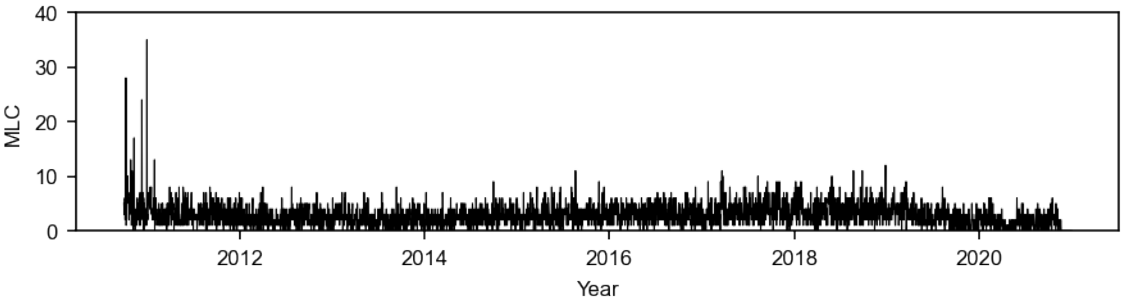

(a)

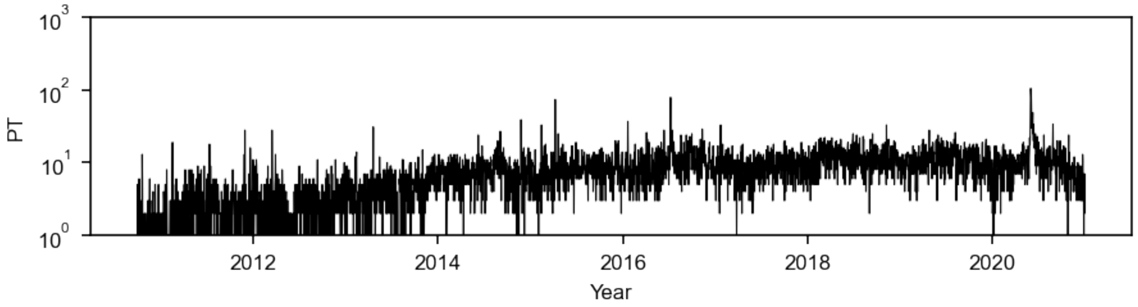

(b)

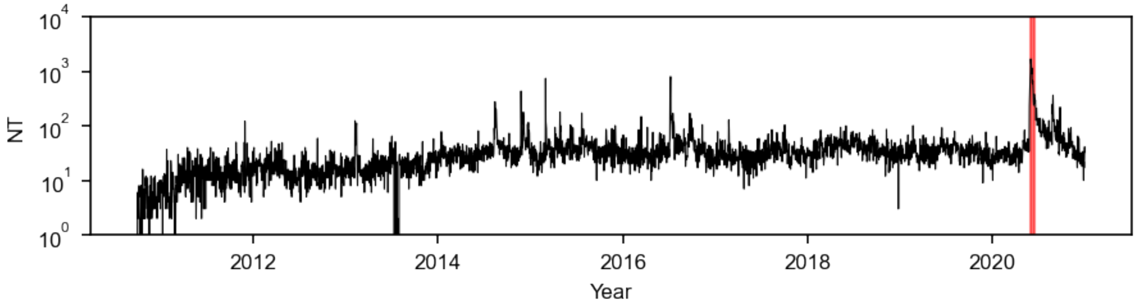

(c)

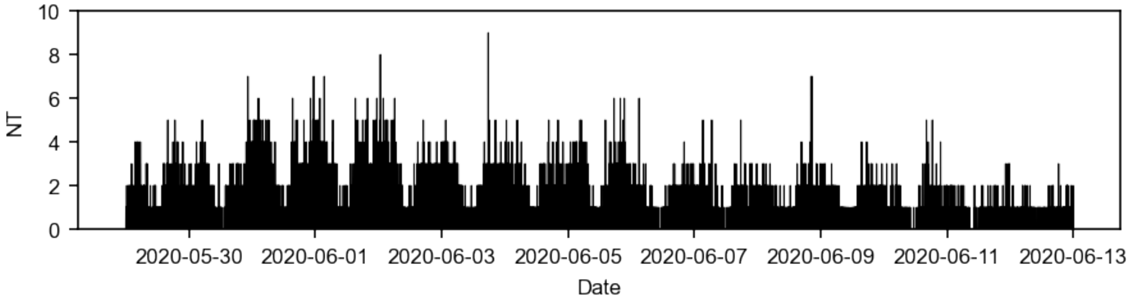

(d)

Supplementary Figure 9: Time series for the Los Angeles metropolitan area. (a) Daily media coverage of local crime (MLC), with a peak of 35 articles registered on December 31, 2010. (b) Daily number of positive tweets (PT) about the police, with a peak of 105 registered on May 31, 2020. (c) Daily number of negative tweets (NT) about the police, with a peak of 1,688 registered on May 31, 2020; activity in the wake of George Floyd's murder is highlighted in red. (d) Zoomed-in view at the resolution of one minute of the number of negative tweets about to the police in the wake of George Floyd's murder period, from May 29, 2020 until June 13, 2020.

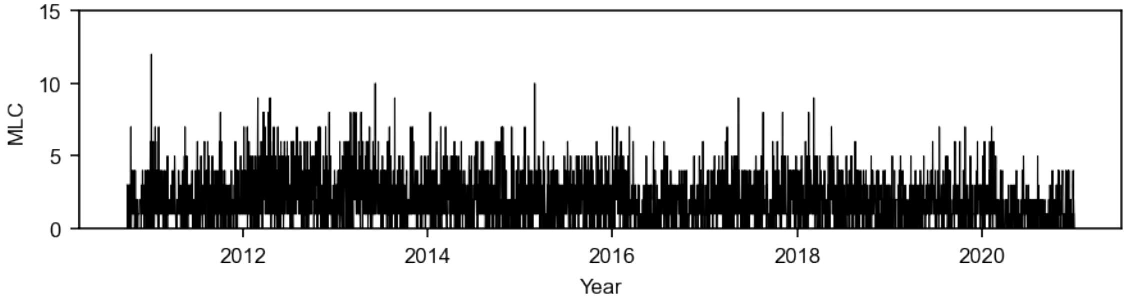

(a)

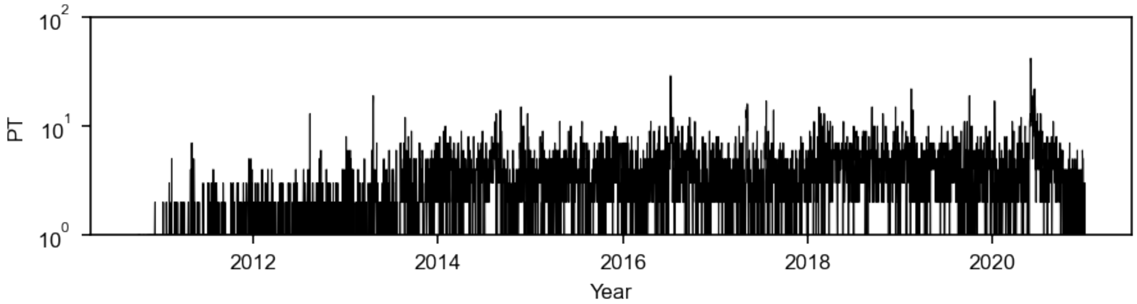

(b)

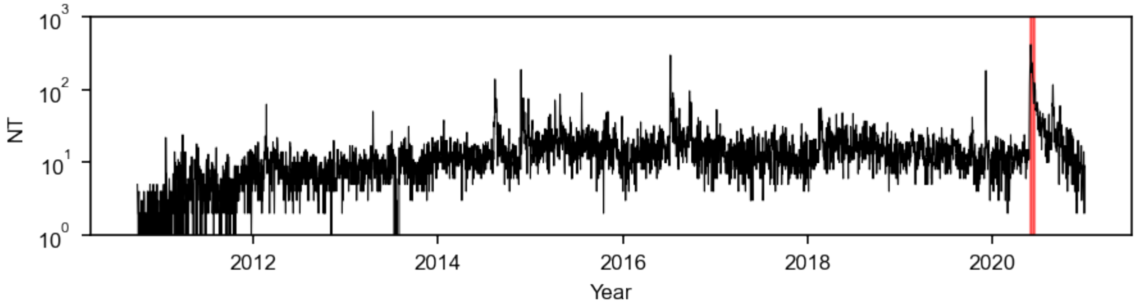

(c)

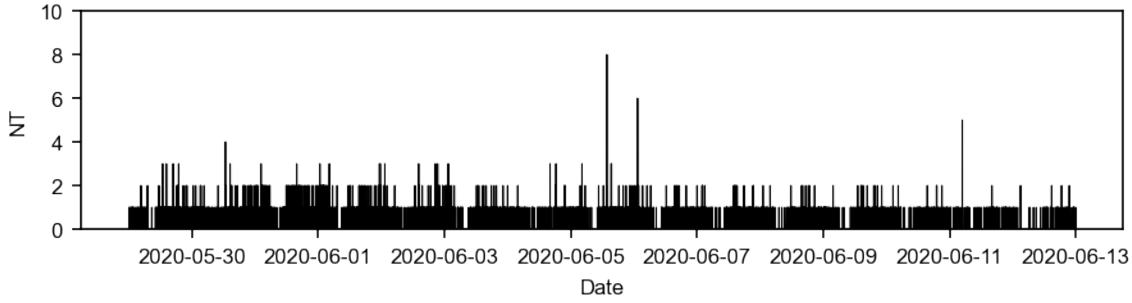

(d)

Supplementary Figure 10: Time series for the Miami metropolitan area. (a) Daily media coverage of local crime (MLC), with a peak of 12 articles registered on January 5, 2011. (b) Daily number of positive tweets (PT) about the police, with a peak of 42 registered on May 31, 2020. (c) Daily number of negative tweets (NT) about the police, with a peak of 415 registered on May 31, 2020; activity in the wake of George Floyd's murder is highlighted in red. (d) Zoomed-in view at the resolution of one minute of the number of negative tweets about to the police in the wake of George Floyd's murder period, from May 29, 2020 until June 13, 2020.

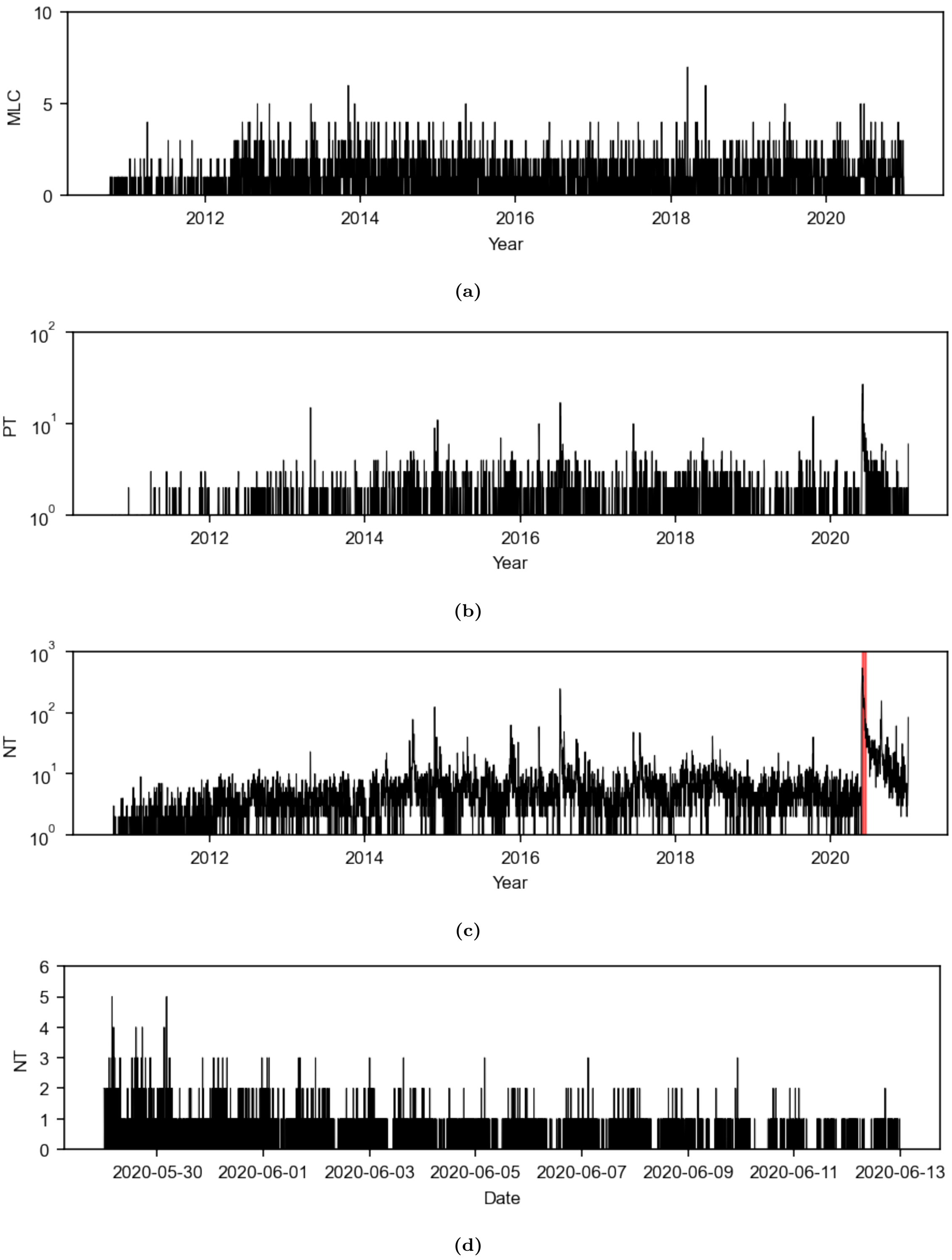

Supplementary Figure 11: Time series for the Minneapolis metropolitan area. (a) Daily media coverage of local crime (MLC), with a peak of 7 articles registered on March 20, 2013. (b) Daily number of positive tweets (PT) about the police, with a peak of 27 registered on May 31, 2020. (c) Daily number of negative tweets (NT) about the police, with a peak of 540 registered on May 29, 2020; activity in the wake of George Floyd's murder is highlighted in red. (d) Zoomed-in view at the resolution of one minute of the number of negative tweets about to the police in the wake of George Floyd's murder period, from May 29, 2020 until June 13, 2020.

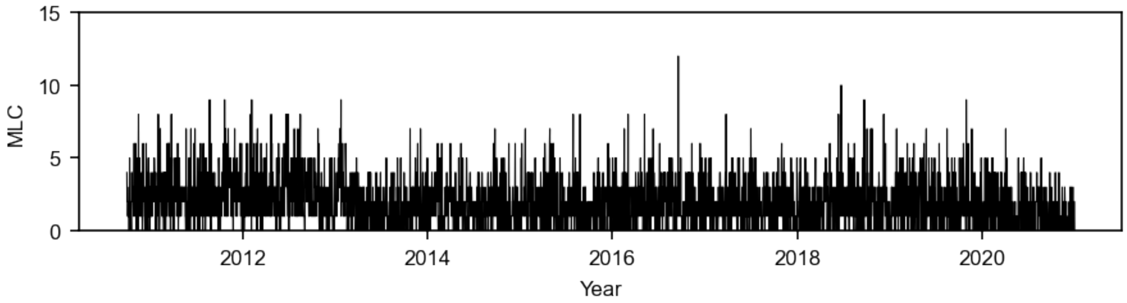

(a)

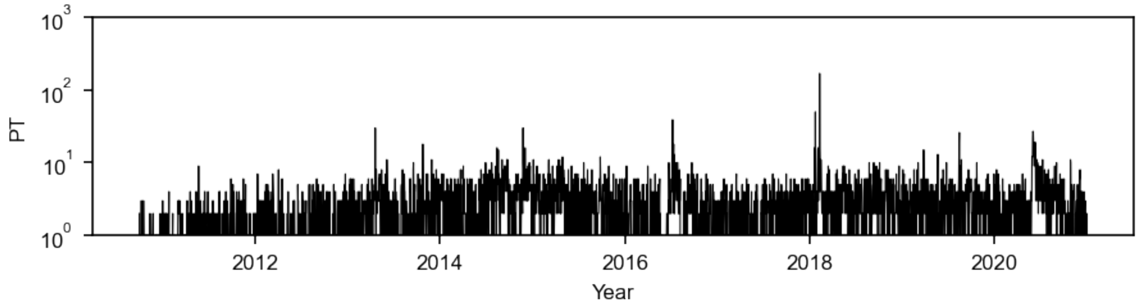

(b)

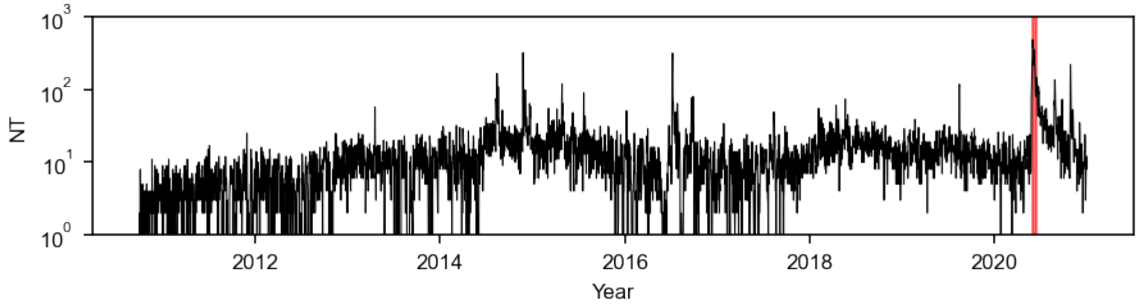

(c)

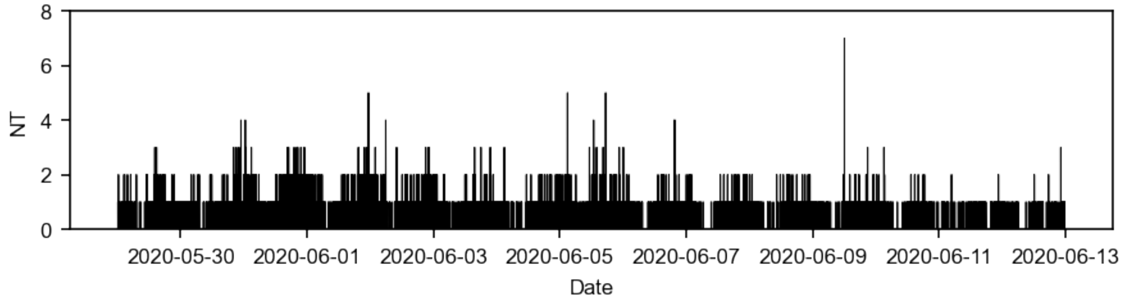

(d)

Supplementary Figure 12: Time series for the Philadelphia metropolitan area. (a) Daily media coverage of local crime (MLC), with a peak of 12 articles registered on September 17, 2016. (b) Daily number of positive tweets (PT) about the police, with a peak of 169 registered on February 9, 2018. (c) Daily number of negative tweets (NT) about the police, with a peak of 487 registered on May 31, 2020; activity in the wake of George Floyd's murder is highlighted in red. (d) Zoomed-in view at the resolution of one minute of the number of negative tweets about to the police in the wake of George Floyd's murder period, from May 29, 2020 until June 13, 2020.

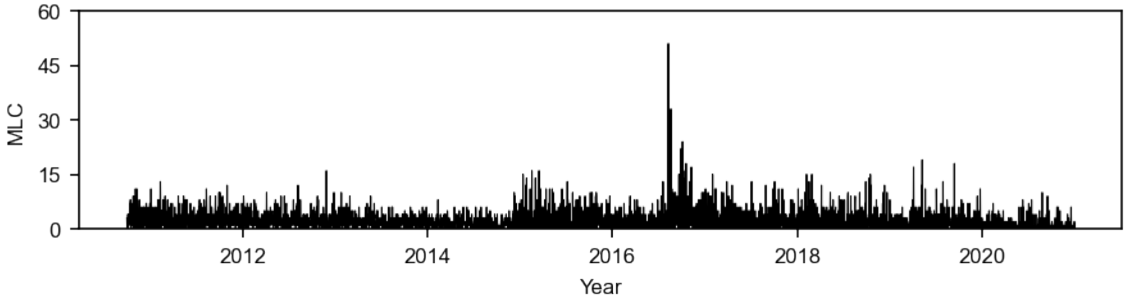

(a)

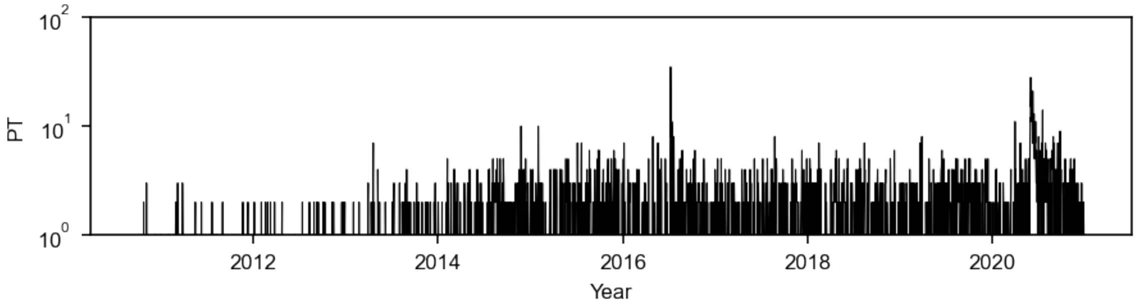

(b)

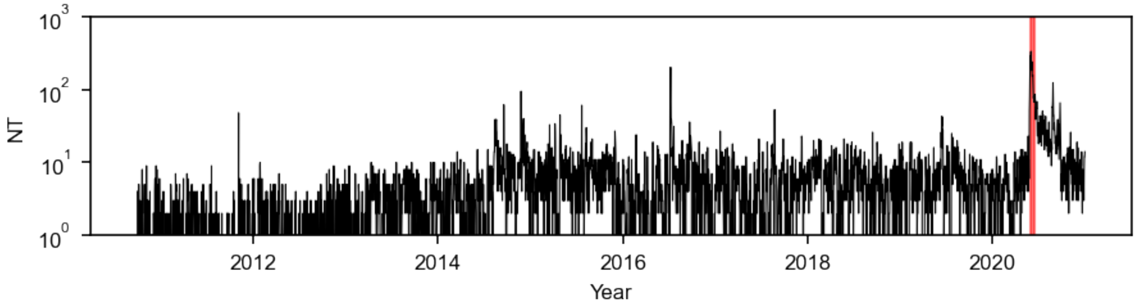

(c)

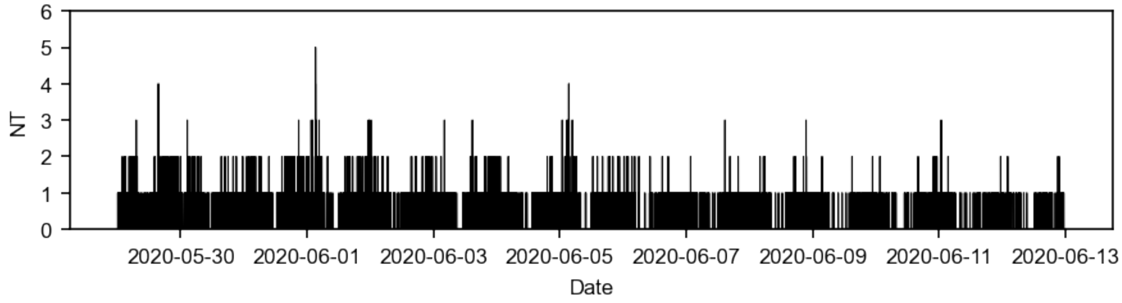

(d)

Supplementary Figure 13: Time series for the Phoenix metropolitan area. (a) Daily media coverage of local crime (MLC), with a peak of 51 articles registered on August 9, 2016. (b) Daily number of positive tweets (PT) about the police, with a peak of 35 registered on July 8, 2016. (c) Daily number of negative tweets (NT) about the police, with a peak of 336 registered on June 1, 2020; activity in the wake of George Floyd's murder is highlighted in red. (d) Zoomed-in view at the resolution of one minute of the number of negative tweets about to the police in the wake of George Floyd's murder period, from May 29, 2020 until June 13, 2020.

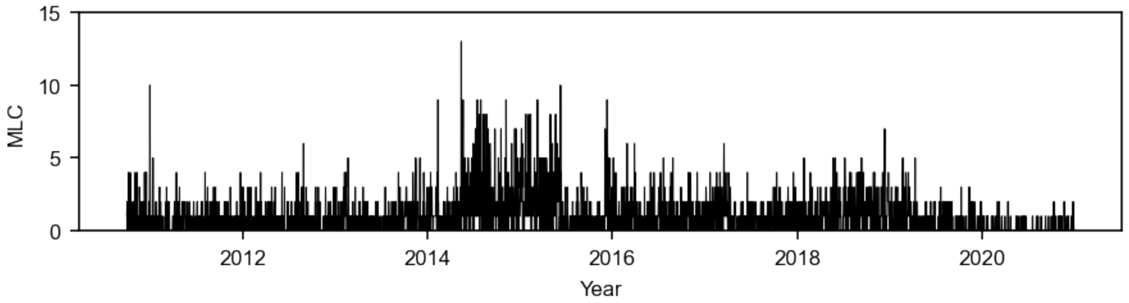

(a)

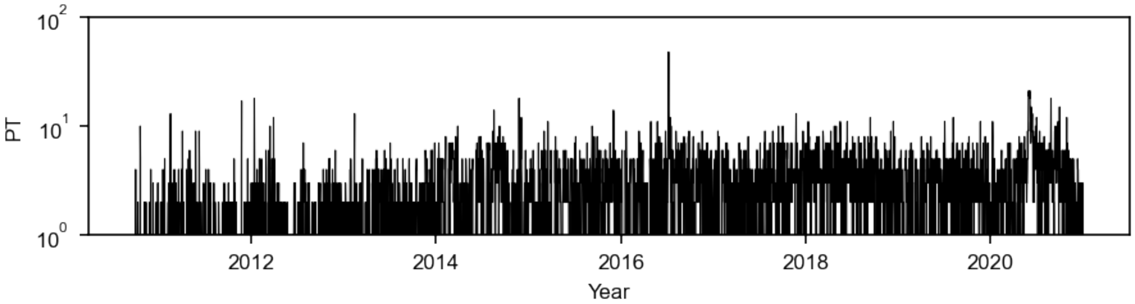

(b)

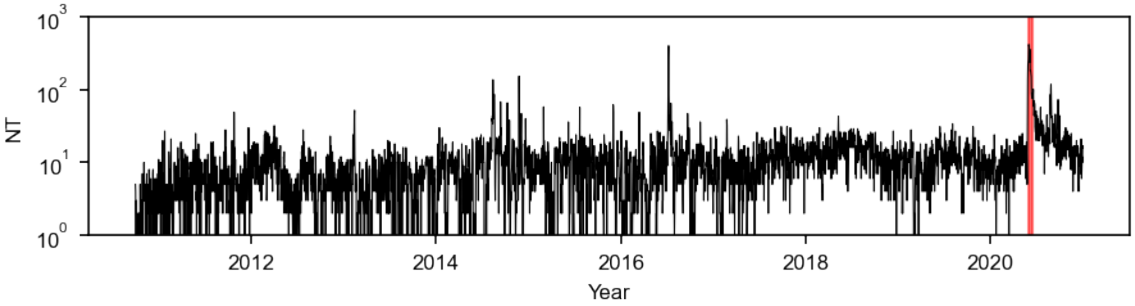

(c)

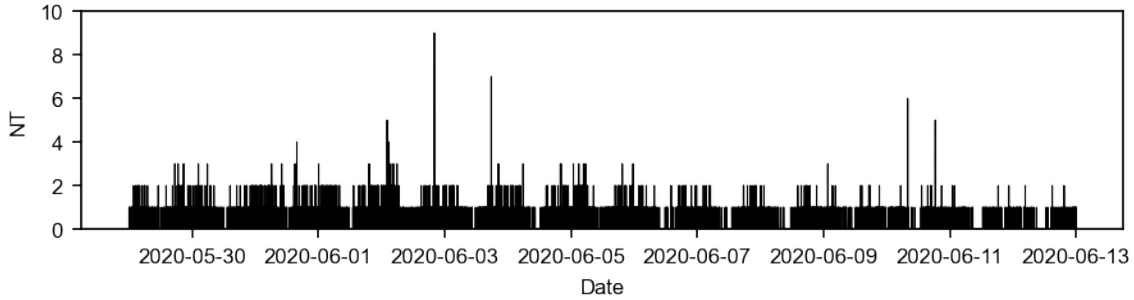

(d)

Supplementary Figure 14: Time series for the Riverside metropolitan area. (a) Daily media coverage of local crime (MLC), with a peak of 13 articles registered on May 14, 2014. (b) Daily number of positive tweets (PT) about the police, with a peak of 48 registered on July 8, 2016. (c) Daily number of negative tweets (NT) about the police, with a peak of 417 registered on May 31, 2020; activity in the wake of George Floyd's murder is highlighted in red. (d) Zoomed-in view at the resolution of one minute of the number of negative tweets about to the police in the wake of George Floyd's murder period, from May 29, 2020 until June 13, 2020.

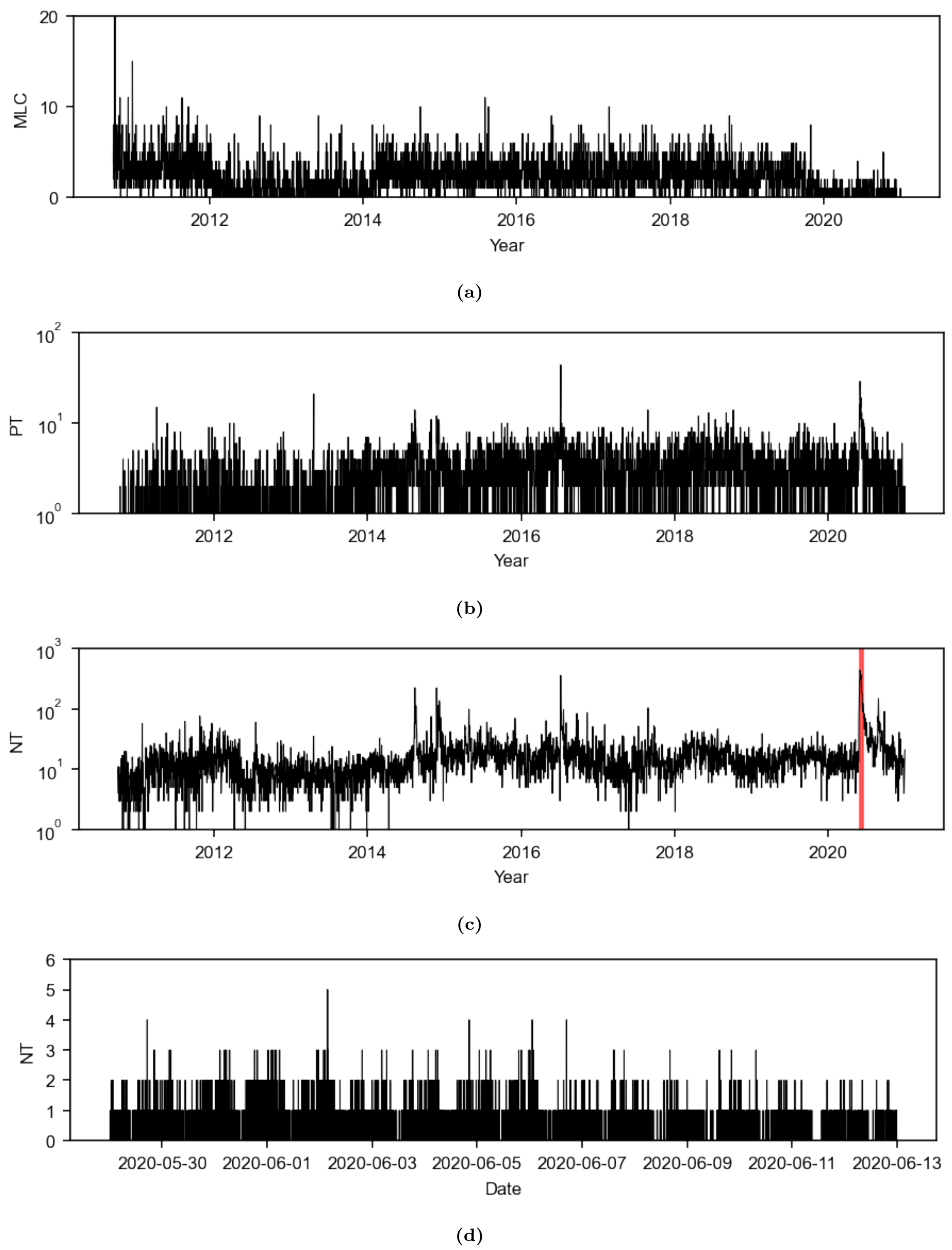

Supplementary Figure 15: Time series for the San Francisco metropolitan area. (a) Daily media coverage of local crime (MLC), with a peak of 20 articles registered on October 9, 2010. (b) Daily number of positive tweets (PT) about the police, with a peak of 44 registered on July 8, 2016. (c) Daily number of negative tweets (NT) about the police, with a peak of 436 registered on May 31, 2020; activity in the wake of George Floyd's murder is highlighted in red. (d) Zoomed-in view at the resolution of one minute of the number of negative tweets about to the police in the wake of George Floyd's murder period, from May 29, 2020 until June 13, 2020.

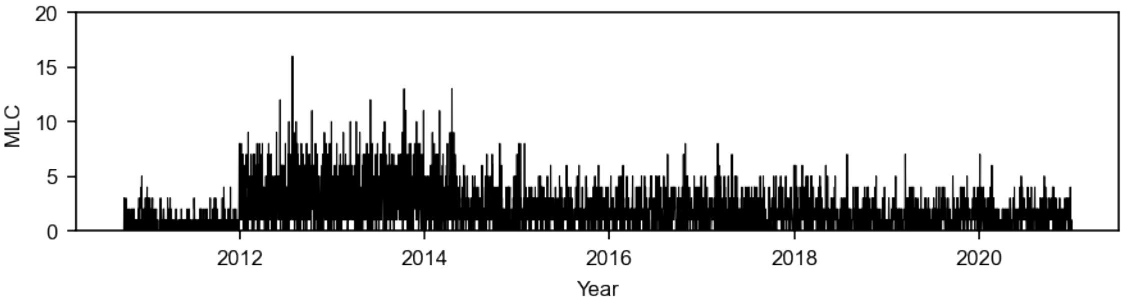

(a)

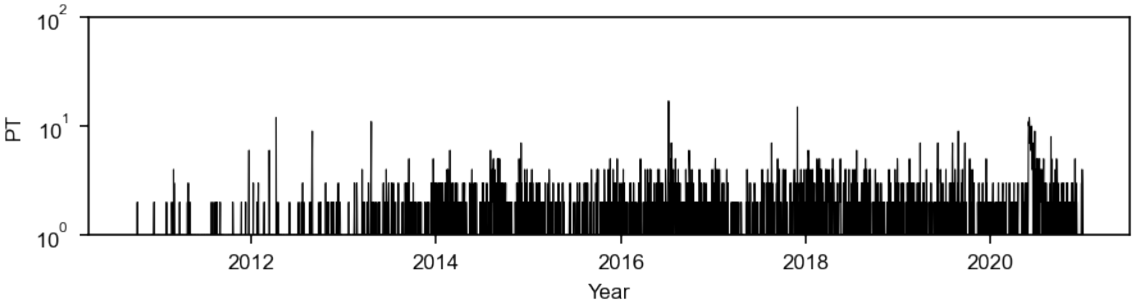

(b)

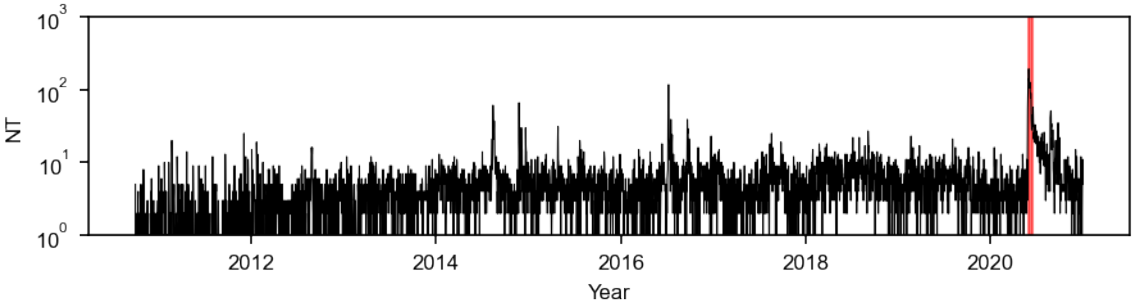

(c)

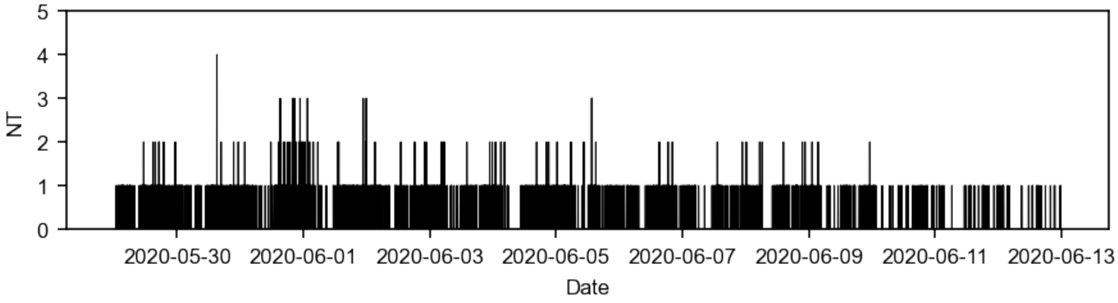

(d)

Supplementary Figure 16: Time series for the Tampa metropolitan area. (a) Daily media coverage of local crime (MLC), with a peak of 16 articles registered on July 28, 2012. (b) Daily number of positive tweets (PT) about the police, with a peak of 17 registered on July 8, 2016. (c) Daily number of negative tweets (NT) about the police, with a peak of 193 registered on May 31, 2020; activity in the wake of George Floyd's murder is highlighted in red. (d) Zoomed-in view at the resolution of one minute of the number of negative tweets about to the police in the wake of George Floyd's murder period, from May 29, 2020 until June 13, 2020.

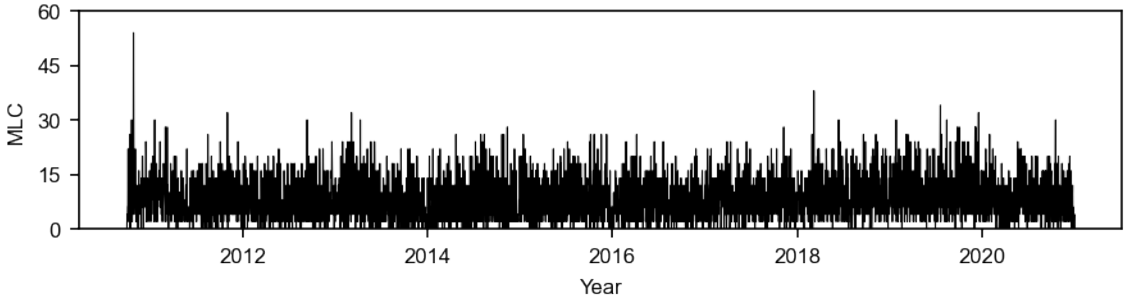

(a)

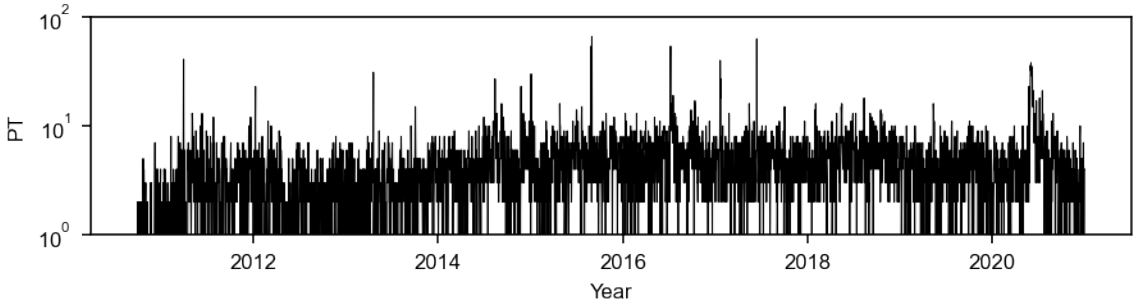

(b)

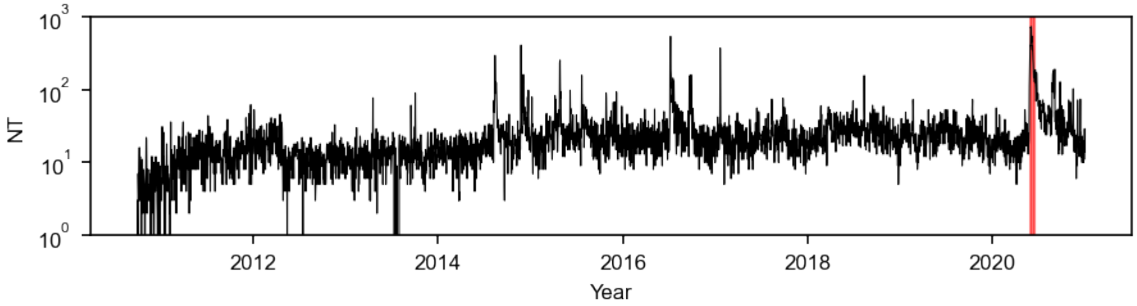

(c)

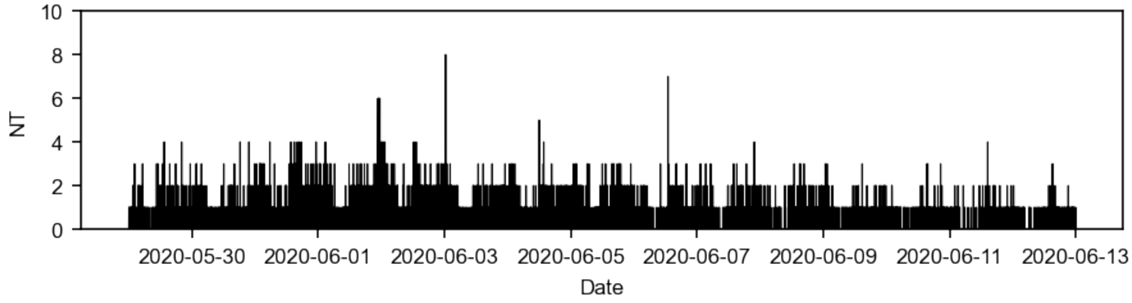

(d)

Supplementary Figure 17: Time series for the Washington, D.C. metropolitan area. (a) Daily media coverage of local crime (MLC), with a peak of 54 articles registered on October 27, 2010. (b) Daily number of positive tweets (PT) about the police, with a peak of 66 registered on September 1, 2015. (c) Daily number of negative tweets (NT) about the police, with a peak of 729 registered on June 2, 2020; activity in the wake of George Floyd's murder is highlighted in red. (d) Zoomed-in view at the resolution of one minute of the number of negative tweets about to the police in the wake of George Floyd's murder period, from May 29, 2020 until June 13, 2020.

Supplementary Note 3 Embedding dimension for analysis with transfer entropy

To ensure that transfer entropy computations are independent of the history of the target variable, one should consider infinitely long time-histories – an unfeasible task given the finiteness of any available time series. To optimize the length of the time-histories we investigated the dependence of the target variables on their past. Specifically, we computed $H(X_t|X_{t-1},...,X_{t-\tau})$ for values of τ ranging from 1 to 4, which is the maximum number allowed for an accurate estimation of the probability mass functions (Silvermanâ€¬, 1988). We found that the conditional entropy values of the target variables (NT and PT) have little to no dependence on τ (Figure 18), suggesting that it is sufficient to perform the analysis using a single time-step in the past (Duan et al., 2013).

Likewise, for a reliable transfer entropy analysis one should assess the influence of longer time-histories for both the source and the conditioning variable. Given the length of our time-series, we could perform such an analysis only for time-histories going two steps into the past. We extended our analysis to the case of two time-histories. Specifically, we examined the following two improved measures, with respect to the source and conditioning variables:

$$TE_{Y\to X|Z} = H(X_{t+1}|X_t, Z_t) - H(X_{t+1}|X_t, Y_t, Y_{t-1}, Z_t) \tag{S1}$$

and

$$TE_{Y\to X|Z} = H(X_{t+1}|X_t, Z_t, Z_{t-1}) - H(X_{t+1}|X_t, Y_t, Z_t, Z_{t-1}). \tag{S2}$$

We statistically tested whether the computed values are different from zero by using the same shuffling scheme described in the main manuscript. In the case where we expanded on the representation of the source (Eq. (S1)), we shuffled the vector $[Y_t, Y_{t-1}]$ all at once to preserve its dynamics with respect to the other variables. Results in Supplementary Tables 2 and 3 are consistent with the conclusions drawn in the main manuscript.

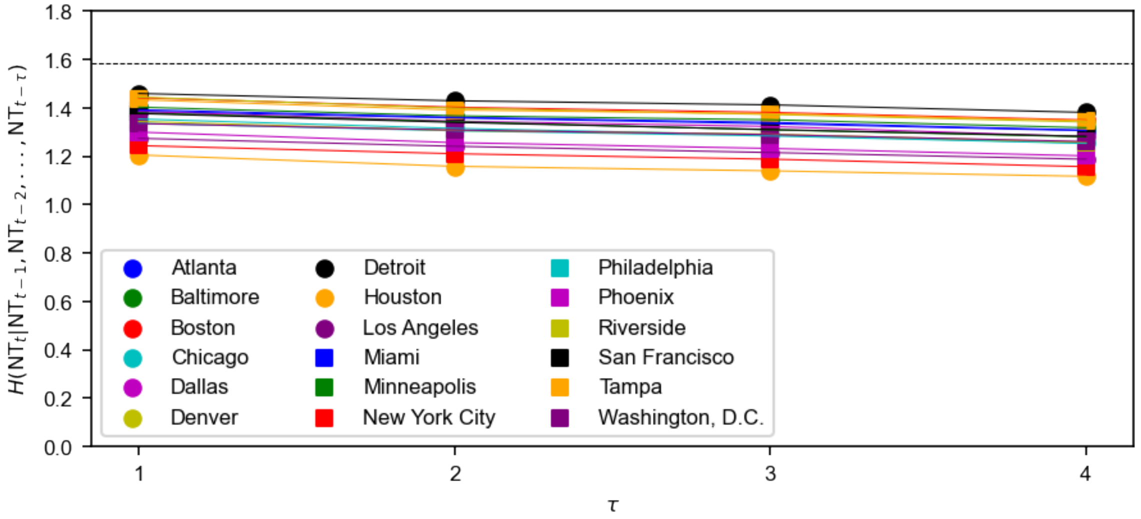

(a)

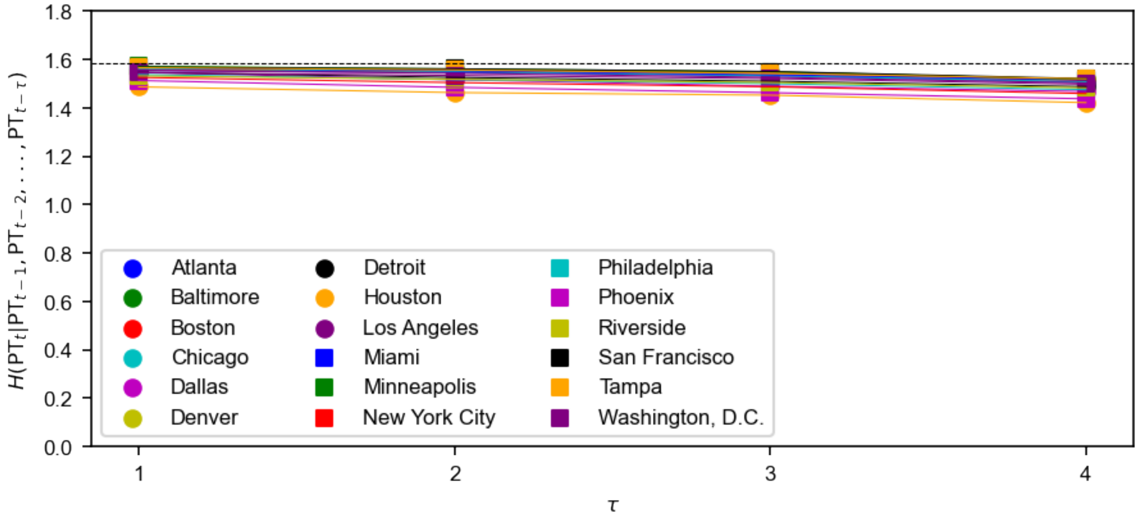

(b)

Supplementary Figure 18: Identification of the appropriate embedding dimension for the target variables for transfer entropy analysis. (a) Number of negative tweets (NT). (b) Number of positive tweets PT. The dashed horizontal line represents the entropy of the time series ($H(\mathrm{NT}_t)$ and $H(\mathrm{PT}_t)$), showing the importance of conditioning on the immediate past.

Metropolitan area	MLC → NT | MPB	MLC → PT | MPB	MPB → NT | MLC
Atlanta	0.0258 (0.0344) $p = 0.7869$	0.0214 (0.0340) $p = 0.9879$	**0.0555** (0.0350) $p < 0.0001$
Baltimore	0.0234 (0.0344) $p = 0.9485$	0.0316 (0.0339) $p = 0.1520$	**0.0446** (0.0349) $p < 0.0001$
Boston	0.0258 (0.0342) $p = 0.7834$	0.0280 (0.0340) $p = 0.5130$	**0.0566** (0.0347) $p < 0.0001$
Chicago	0.0338 (0.0346) $p = 0.0761$	0.0304 (0.0340) $p = 0.2578$	**0.0575** (0.0351) $p < 0.0001$
Dallas	**0.0376** (0.0344) $p = 0.0076$	0.0296 (0.0340) $p = 0.3377$	**0.0620** (0.0350) $p < 0.0001$
Denver	0.0309 (0.0341) $p = 0.2304$	0.0249 (0.0339) $p = 0.8426$	**0.0669** (0.0347) $p < 0.0001$
Detroit	0.0311 (0.0342) $p = 0.2072$	0.0289 (0.0341) $p = 0.4138$	**0.0487** (0.0348) $p < 0.0001$
Houston	0.0288 (0.0351) $p = 0.5549$	0.0316 (0.0340) $p = 0.1632$	**0.0519** (0.0354) $p < 0.0001$
Los Angeles	**0.0352** (0.0348) $p = 0.0419$	**0.0368** (0.0340) $p = 0.0094$	**0.0539** (0.0353) $p < 0.0001$
Miami	0.0283 (0.0343) $p = 0.5165$	0.0278 (0.0341) $p = 0.5389$	**0.0557** (0.0350) $p < 0.0001$
Minneapolis	0.0271 (0.0344) $p = 0.6644$	0.0273 (0.0341) $p = 0.6049$	**0.0569** (0.0351) $p < 0.0001$
New York	0.0325 (0.0352) $p = 0.1772$	0.0290 (0.0341) $p = 0.4022$	**0.0596** (0.0354) $p < 0.0001$
Philadelphia	0.0249 (0.0346) $p = 0.08716$	0.0320 (0.0339) $p = 0.1304$	**0.0537** (0.0351) $p < 0.0001$
Phoenix	0.0201 (0.0347) $p = 0.9975$	0.0300 (0.0341) $p = 0.2996$	**0.0674** (0.0354) $p < 0.0001$
Riverside	0.0304 (0.0345) $p = 0.3016$	0.0274 (0.0341) $p = 0.5946$	**0.0472** (0.0351) $p < 0.0001$
San Francisco	0.0312 (0.0344) $p = 0.2152$	0.0230 (0.0338) $p = 0.9473$	**0.0545** (0.0349) $p < 0.0001$
Tampa	0.0277 (0.0343) $p = 0.5820$	0.0319 (0.0341) $p = 0.1471$	**0.0718** (0.0348) $p < 0.0001$
Washington, D.C.	0.0329 (0.0347) $p = 0.1185$	0.0300 (0.0340) $p = 0.2891$	**0.0605** (0.0351) $p < 0.0001$

Supplementary Table 2: Conditional transfer entropy results for the 18 chosen metropolitan areas considering two time-steps in the past for the source variable (Eq. (S1)). The top left number in each cell of the table represents the conditional transfer entropy value. Numbers in parentheses reflect the 95% quantile of a surrogate distribution obtained from a permutation test. The p-values indicate the significance of the same test. Bold values indicate a significant conditional transfer entropy at $\alpha = 0.05$.

Metropolitan area	MLC → NT | MPB	MLC → PT | MPB	MPB → NT | MLC
Atlanta	0.0216 (0.0271) $p = 0.5201$	0.0244 (0.0267) $p = 0.1726$	**0.0445** (0.0268) $p < 0.0001$
Baltimore	0.0199 (0.0270) $p = 0.7394$	**0.0273** (0.0267) $p = 0.0348$	**0.0303** (0.0268) $p = 0.0033$
Boston	0.0187 (0.0268) $p = 0.8525$	0.0192 (0.0266) $p = 0.7826$	**0.0428** (0.0266) $p < 0.0001$
Chicago	0.0214 (0.0272) $p = 0.5643$	0.0240 (0.0266) $p = 0.1949$	**0.0515** (0.0270) $p < 0.0001$
Dallas	0.0251 (0.0271) $p = 0.1479$	0.0222 (0.0266) $p = 0.3929$	**0.0546** (0.0268) $p < 0.0001$
Denver	0.0260 (0.0269) $p = 0.0833$	0.0168 (0.0267) $p = 0.9532$	**0.0505** (0.0266) $p < 0.0001$
Detroit	0.0217 (0.0269) $p = 0.4895$	0.0195 (0.0267) $p = 0.7628$	**0.0433** (0.0266) $p < 0.0001$
Houston	**0.0296** (0.0270) $p = 0.0070$	0.0222 (0.0269) $p = 0.4247$	**0.0323** (0.0275) $p = 0.0018$
Los Angeles	0.0189 (0.0275) $p = 0.8773$	0.0265 (0.0268) $p = 0.0587$	**0.0508** (0.0272) $p < 0.0001$
Miami	0.0191 (0.0269) $p = 0.8122$	0.0162 (0.0268) $p = 0.9766$	**0.0497** (0.0268) $p < 0.0001$
Minneapolis	0.01968 (0.0271) $p = 0.7810$	0.0244 (0.0266) $p = 0.1588$	**0.0459** (0.0269) $p < 0.0001$
New York	0.0274 (0.0277) $p = 0.0599$	0.0188 (0.0268) $p = 0.8263$	**0.0436** (0.0274) $p < 0.0001$
Philadelphia	0.0193 (0.0272) $p = 0.8143$	0.0185 (0.0266) $p = 0.8545$	**0.0426** (0.0270) $p < 0.0001$
Phoenix	0.0199 (0.0274) $p = 0.7811$	0.0202 (0.0268) $p = 0.6782$	**0.0467** (0.0272) $p < 0.0001$
Riverside	0.01901 (0.0271) $p = 0.8378$	0.0201 (0.0267) $p = 0.6862$	**0.0359** (0.0269) $p < 0.0001$
San Francisco	0.0181 (0.0271) $p = 0.9085$	0.0198 (0.0266) $p = 0.7204$	**0.0444** (0.0269) $p < 0.0001$
Tampa	0.0250 (0.0268) $p = 0.1353$	0.0165 (0.0266) $p = 0.9654$	**0.0497** (0.0267) $p < 0.0001$
Washington, D.C.	0.0242 (0.0273) $p = 0.2320$	0.0243 (0.0267) $p = 0.1739$	**0.0549** (0.0272) $p < 0.0001$

Supplementary Table 3: Conditional transfer entropy results for the 18 chosen metropolitan areas considering two time-steps in the past for the conditioning variable (Eq. (S2)). The top left number in each cell of the table represents the conditional transfer entropy value. Numbers in parentheses reflect the 95% quantile of a surrogate distribution obtained from a permutation test. The p-values indicate the significance of the same test. Bold values indicate a significant conditional transfer entropy at $\alpha = 0.05$.

Supplementary Note 4 Transfer entropy analysis with general sentiment analysis

While aspect-based sentiment analysis is the state-of-the-art for stance analysis in NLP, the Vader dictionary has been the method of choice in sentiment analysis for many years. We performed the transfer entropy analysis presented in the main manuscript using the `Natural Language Toolkit` (NLTK) package by Bird et al. (2009), which relies on the Vader dictionary (Hutto & Gilbert, 2015). NLTK is a powerful tool built on machine learning algorithms to obtain a compounded sentiment based on individual words and their relative

positions in a piece of text. The output score of NLTK determined how positive or negative an assertion is within a range of $[-1, 1]$. In our analysis, tweets with NLTK scores above 0 were classified as positive, and tweets with scores below 0 were classified as negative.

Results of the statistical analysis for all metropolitan areas (Supplementary Table 4) offer strong support in favor of H2 (the null hypothesis of independence was always rejected). Limited support in favor of H1a was gathered for the metropolitan areas of Los Angeles and Riverside (the null hypothesis of independence was not rejected for all the other 16 metropolitan areas), while no support was obtained in favor of H1b (the null hypothesis of independence was never rejected).

Metropolitan area	MLC → NT | MPB	MLC → PT | MPB	MPB → NT | MLC
Atlanta	0.0058 (0.0099) $p = 0.7296$	0.0074 (0.0099) $p = 0.3630$	**0.0257** (0.0100) $p < 0.0001$
Baltimore	0.0059 (0.0099) $p = 0.7296$	0.0070 (0.0099) $p = 0.4598$	**0.0109** (0.0099) $p = 0.0188$
Boston	0.0042 (0.0099) $p = 0.9700$	0.0060 (0.0099) $p = 0.7024$	**0.0192** (0.0099) $p < 0.0001$
Chicago	0.0070 (0.0100) $p = 0.4683$	0.0071 (0.0099) $p = 0.4340$	**0.0235** (0.0099) $p < 0.0001$
Dallas	0.0061 (0.0099) $p = 0.6820$	0.0073 (0.0099) $p = 0.3994$	**0.0379** (0.0099) $p < 0.0001$
Denver	0.0075 (0.0099) $p = 0.3640$	0.0083 (0.0099) $p = 0.2069$	**0.0237** (0.0100) $p < 0.0001$
Detroit	0.0063 (0.0099) $p = 0.6336$	0.0067 (0.0099) $p = 0.5391$	**0.0247** (0.0099) $p < 0.0001$
Houston	0.0084 (0.0102) $p = 0.2127$	0.0060 (0.0100) $p = 0.7170$	**0.0146** (0.0101) $p = 0.0001$
Los Angeles	**0.0104** (0.0100) $p = 0.0325$	0.0072 (0.0102) $p = 0.4587$	**0.0304** (0.0100) $p < 0.0001$
Miami	0.0070 (0.0099) $p = 0.4744$	0.0039 (0.0099) $p = 0.9862$	**0.0362** (0.0099) $p < 0.0001$
Minneapolis	0.0098 (0.0099) $p = 0.0546$	0.0094 (0.0099) $p = 0.0804$	**0.0376** (0.0099) $p < 0.0001$
New York	0.0066 (0.0100) $p = 0.5801$	0.0091 (0.0099) $p = 0.1046$	**0.0266** (0.0100) $p < 0.0001$
Philadelphia	0.0070 (0.0099) $p = 0.4793$	0.0063 (0.0099) $p = 0.6381$	**0.0242** (0.0100) $p < 0.0001$
Phoenix	0.0075 (0.0099) $p = 0.3608$	0.0079 (0.0099) $p = 0.2810$	**0.0245** (0.0100) $p < 0.0001$
Riverside	**0.0101** (0.0099) $p = 0.0407$	0.0072 (0.0099) $p = 0.4124$	**0.0240** (0.0100) $p < 0.0001$
San Francisco	0.0068 (0.0100) $p = 0.5152$	0.0061 (0.0099) $p = 0.6859$	**0.0210** (0.0100) $p < 0.0001$
Tampa	0.0048 (0.0099) $p = 0.9257$	0.0083 (0.0099) $p = 0.2025$	**0.0323** (0.0100) $p < 0.0001$
Washington, D.C.	0.0068 (0.0099) $p = 0.5237$	0.0089 (0.0099) $p = 0.1218$	**0.0242** (0.0099) $p < 0.0001$

Supplementary Table 4: Conditional transfer entropy results for the 18 chosen metropolitan areas. The top left number in each cell of the table represents the conditional transfer entropy value. Numbers in parentheses reflect the 95% quantile of a surrogate distribution obtained from a permutation test. The p-values indicate the significance of the same test. Bold values indicate a significant conditional transfer entropy at $\alpha = 0.05$.

Supplementary Note 5 Transfer entropy analysis with local crimes, rather than their media coverage

In the main manuscript, we studied the role of the number of articles from local newspapers mentioning crime in the metropolitan area (media coverage of local crime) in the formation of public sentiment towards the police. However, media coverage of crimes may not accurately capture the perception of local crimes. For example, Beckett (1999) suggests that this representation is not always true, while Enns (2016) points out that public opinion can be more strongly shaped by objective crime rates. Here, we explore whether our claims are robust with respect to the use of objective measures of crime.

To this end, we obtained data on the number of crimes in New York City from the Open Data Portal (New York City Government, 2021). This database contains records of crimes divided into three categories: felonies, violations, and misdemeanors. The crimes are further grouped into 72 sub-categories, with 31 sub-categories of felonies, 15 of violations, and 40 of misdemeanors. Thirteen sub-categories were included in multiple categories. We collected data on crimes from October 1, 2010 to December 31, 2020 and focused only on crimes classified as felonies, resulting in a total count of $1,500,231$ crimes (Supplementary Figure 20a). Geo-located tweets were scraped as described in the main manuscript; however, instead of scraping tweets from the whole New York City metropolitan area, we scraped tweets only where there was overlap with the available crime data (in addition to a border region that accounts for regular commutes to the city, see Supplementary Figures 19, 20b, and 20c). The keywords considered were "police," "cop," and "NYPD."

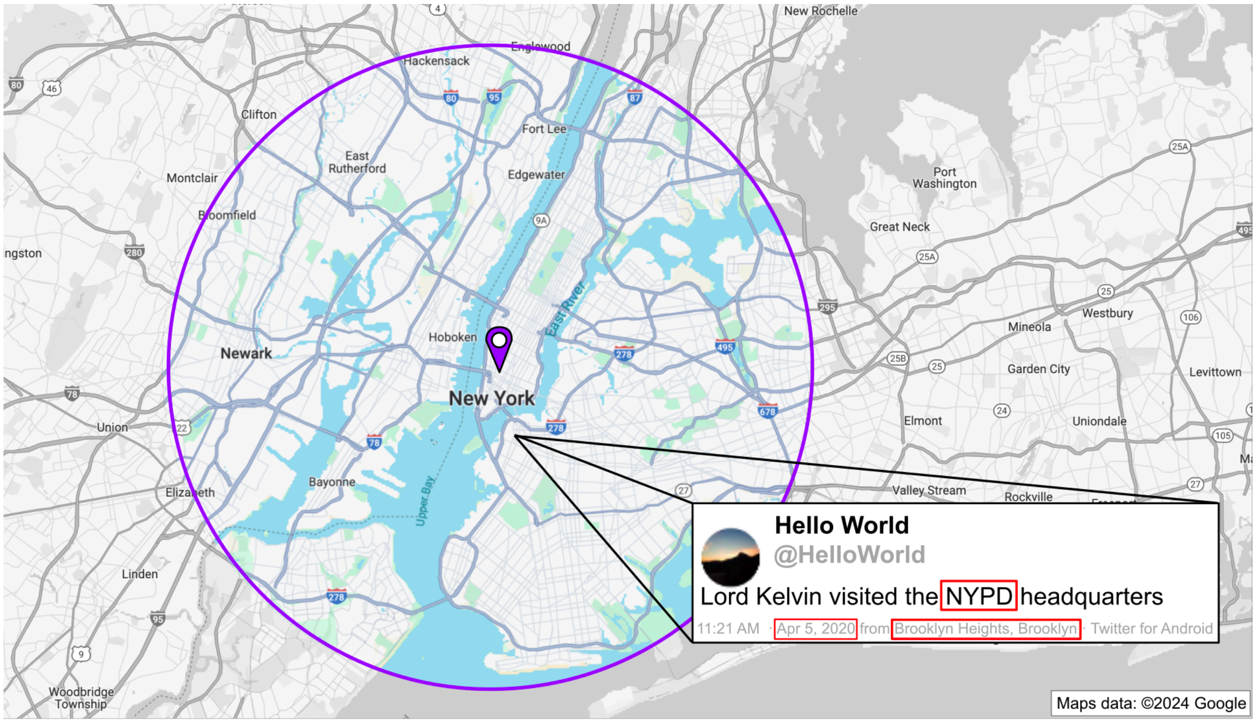

Supplementary Figure 19: Collection of geo-located tweets. The shaded blue area represents the region from which tweets were collected, with the pinpoint at its center. An example of a geo-located tweet is displayed on the bottom right. The date, location, and keyword of the tweet are highlighted in red. The tweet presented in this illustrative figure is fake.

In addition to the raw count of crimes, we constructed a proxy of perception of local crimes that acknowledges that different crimes could be differently perceived by residents (Roberts & Stalans, 2018; Moreno-Vera et al., 2021). In this vein, we considered a weighting scheme for crimes based on the National Survey of Crime Severity (NSCS) from the Bureau of Justice Statistics (Wolfgang, 1985). NSCS is the most detailed and comprehensive survey performed on the topic, where the authors aimed to assess how the public perceives

different crimes. A total of $51,623$ adults were asked to rate the severity of 204 different crimes relative to the severity of theft of a bicycle. While bicycle theft was scored at a benchmark value of 1, teenagers skipping school received the lowest score of 0.2, and planting a bomb in a public building causing the deaths of 20 people obtained the maximum score of 72. We manually matched the 31 felonies reported by New York City's Open Data Portal with NSCS's severity scores. In cases where a felony could possibly be matched with multiple scores, the average of the scores was taken. Once the severity score of each felony was obtained, we summed the severity scores for all felonies in a given day and obtained a weighted count of the daily crimes (Supplementary Figure 20d).

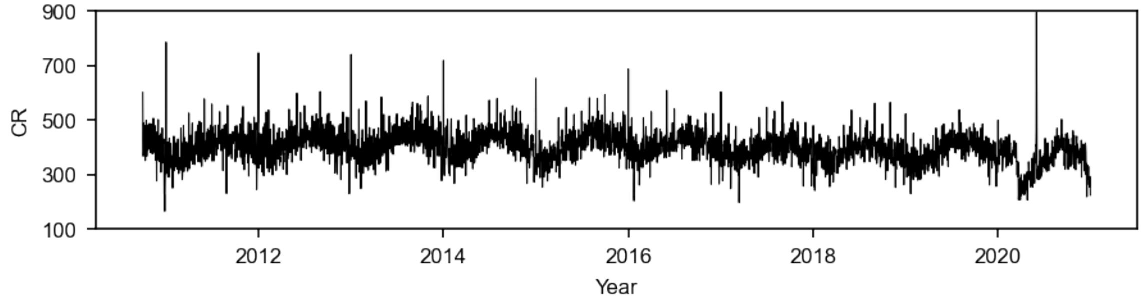

(a)

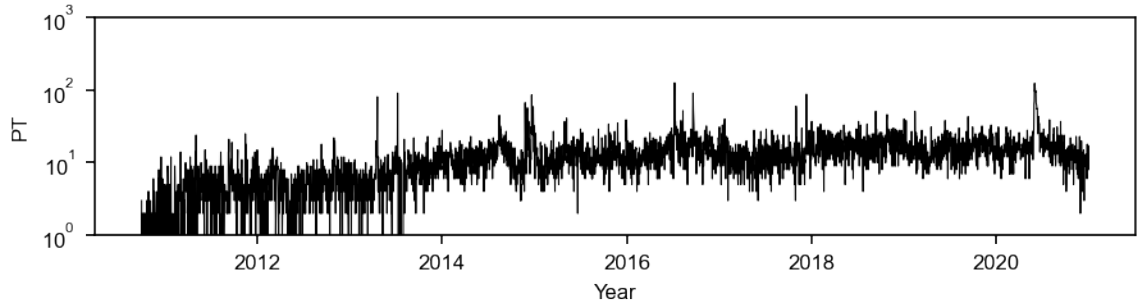

(b)

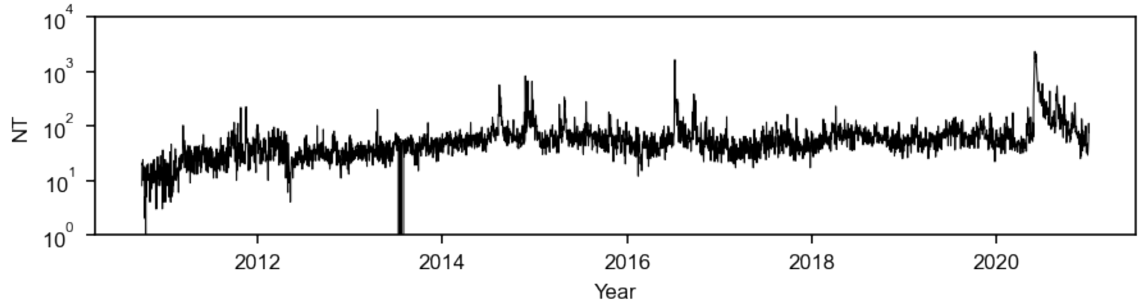

(c)

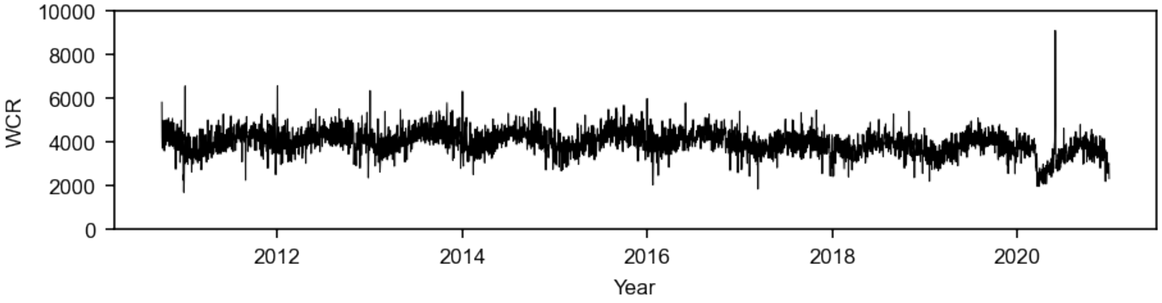

(d)

Supplementary Figure 20: Time series for New York City for the study of local crimes (not their media coverage). (a) Daily number of crimes (CR), with a peak of 895 crimes registered on June 1, 2020. (b) Daily number of positive tweets (PT) about the police, with a peak of 126 registered on July 8, 2016. (c) Daily number of negative tweets (NT) about the police, with a peak of 2312 registered on May 31, 2020. (d) Daily weighted number of crimes (WCR) about the police, with a peak of 9102.03 registered on June 1, 2020.

PLC in New York City	LC $\rightarrow$ NT | MPB	LC $\rightarrow$ PT | MPB	MPB $\rightarrow$ NT | LC
Number of local crimes	0.0058 (0.0100)<br>$p = 0.7691$	0.0084 (0.0099)<br>$p = 0.1958$	**0.0295** (0.0100)<br>$p < 0.0001$
Weighted number of local crimes	0.0043 (0.0100)<br>$p = 0.9668$	0.0085 (0.0099)<br>$p = 0.1765$	**0.0283** (0.0101)<br>$p < 0.0001$

Supplementary Table 5: Conditional transfer entropy results for New York City for local crimes (LC), not their media coverage. The top left number in each cell of the table represents the conditional transfer entropy value. Numbers in parentheses are the 95% quantile of a surrogate distribution obtained from a permutation test. The p-values indicate the significance of the same test. Bold values identify a significant conditional transfer entropy at $\alpha = 0.05$.

We performed the same transfer entropy analysis as in the main manuscript by replacing media coverage of local crime with either the number of local crimes or the weighted number of local crimes (Supplementary Table 5). In both cases, causal relationships from local crimes to the number of positive or negative tweets failed to reach statistical significance. Importantly, the influence of media coverage of police brutality on the number of negative tweets was not affected by the use of local crimes; whether we used socially constructed (media coverage of local crime) or an objective (number of local crimes and weighted number of local crimes) measure of crime, H2 holds.

Supplementary References

Beckett, K. (1999). *Making crime pay: Law and order in contemporary american politics.* Oxford University Press.

Bird, S., Klein, E., & Loper, E. (2009). *Natural language processing with Python: analyzing text with the natural language toolkit.* O'Reilly Media, Inc.

Cision. (2018). *Top 10 U.S. daily newspapers on Twitter.* (https://www.cision.com/2018/01/top-10-u-s-daily-newspapers-on-twitter/)

Duan, P., Yang, F., Chen, T., & Shah, S. L. (2013). Direct causality detection via the transfer entropy approach. *IEEE Transactions on Control Systems Technology*, *21*(6), 2052–2066.

Enns, P. K. (2016). *Incarceration nation.* Cambridge University Press.

Hutto, C., & Gilbert, E. (2015). Vader: A parsimonious rule-based model for sentiment analysis of social media text. In *Proceedings of the 8th International Conference on Weblogs and Social Media.*

Moreno-Vera, F., Lavi, B., & Poco, J. (2021). Urban perception: Can we understand why a street is safe? In *Mexican International Conference on Artificial Intelligence* (pp. 277–288).

New York City Government. (2021). *NYC Open Data.* (https://opendata.cityofnewyork.us/)

OfficialUSA. (2022). *USA newspapers.* (https://www.officialusa.com/stateguides/media/newspapers/)

Roberts, J. V., & Stalans, L. J. (2018). *Public opinion, crime, and criminal justice.* Routledge.

Silvermanâ€¬, â. W. (1988). *Density estimation for statistics and data analysis. chapman & hall, London–New York 1986.* Wiley Online Library.

Turvill, W. (2018). *US newspaper circulations: Americaâ€™s top 25 titles have lost 30% of print sales in two years.* (https://pressgazette.co.uk/us-newspaper-circulations/)

Wolfgang, M. E. (1985). *The national survey of crime severity.* US Department of Justice, Bureau of Justice Statistics.
